# Evaluation of Swab-Seq as a scalable, sensitive assay for community surveillance of SARS-CoV-2 infection

**DOI:** 10.1038/s41598-022-06901-5

**Published:** 2022-02-23

**Authors:** HyunJin Kang, Sheilah Allison, Amber Spangenberg, Tara Carr, Ryan Sprissler, Marilyn Halonen, Darren A. Cusanovich

**Affiliations:** 1 Asthma and Airway Disease Research Center (A2DRC), University of Arizona, Tucson, AZ USA; 2 Center for Applied Genetics and Genomic Medicine, University of Arizona, Tucson, AZ USA; 3 University of Arizona Genetics Core, University of Arizona, Tucson, AZ USA; 4 Department of Cellular and Molecular Medicine, University of Arizona, Tucson, AZ USA; 5 BIO5 Institute, University of Arizona, Tucson, AZ USA

**Keywords:** RNA sequencing, Viral infection

## Abstract

The ongoing SARS-CoV-2 pandemic and subsequent demand for viral testing has led to issues in scaling diagnostic lab efforts and in securing basic supplies for collection and processing of samples. This has motivated efforts by the scientific community to establish improved protocols that are more scalable, less resource intensive, and less expensive. One such developmental effort has resulted in an assay called “Swab-Seq”, so named because it was originally developed to work with dry nasal swab samples. The existing gold standard test consists of RNA extracted from a nasopharyngeal (NP) swab that is subjected to quantitative reverse transcription polymerase chain reaction (qRT-PCR). Swab-Seq adapts this method to a next-generation sequencing readout. By pairing this modification with extraction-free sampling techniques, Swab-Seq achieves high scalability, low cost per sample, and a reasonable turnaround time. We evaluated the effectiveness of this assay in a community surveillance setting by testing samples collected from both symptomatic and asymptomatic individuals using the traditional NP swab. In addition, we evaluated extraction-free sampling techniques (both saliva and saline mouth gargle samples). We found the assay to be as clinically sensitive as the qRT-PCR assay, adaptable to multiple sample types, and able to easily accommodate hundreds of samples at a time. We thus provide independent validation of Swab-Seq and extend its utility regarding sample type and sample stability. Assays of this type greatly expand the possibility of routine, noninvasive, repeated testing of asymptomatic individuals suitable for current and potential future needs.

## Introduction

As the SARS-CoV-2 virus was transmitted across the world in late 2019 and early 2020, information about the virus spread almost as swiftly. While the initial cases were first reported outside of China on December 30 of 2019^1^, the full genome of the virus was deposited in GenBank by January 5 of 2020 (accession number MN908947^2^). Impressively, by January 13, the World Health Organization (WHO) had already released a quantitative reverse-transcriptase polymerase chain reaction (qRT-PCR) assay, which was developed using portions of SARS-CoV-2 sequence that had previously been released to create a set of three different probe/primer pairs^3^.

While many additional testing strategies have been developed, the most common sample collection workflow for testing for the presence of the virus involves a nasopharyngeal (NP) swab administered by a healthcare provider, placed in transport media and transferred to a diagnostic laboratory^4^. At that point, RNA is extracted from the media and used as a template for a qRT-PCR test. Several alternative sampling techniques have been tested^5^, however the Center for Disease Control (CDC) and WHO have listed NP swabs as the primary recommended sampling technique throughout the pandemic^6,7^. Nonetheless, the ideal sampling type remains unresolved^4,8–11^. Significantly increased demand for testing expanded rapidly throughout 2020, leading to worldwide shortages of many components necessary for this workflow. And these shortages in turn motivated many groups to implement alternative existing methods (e.g. Ref.^12^) or to develop protocols that eliminated many of the standard steps (e.g. Refs.^4,13–15^). While various new assays have reported a wide range of times to return results, scalability, and sensitivity, many groups have emphasized that the most important feature of an assay is that it is sufficiently scalable to allow for repeated testing of asymptomatic populations^16–18^.

One such assay, called “Swab-Seq”, has been developed primarily by groups at UCLA and Octant Bio^14^. The Swab-Seq assay uses a set of primers targeting the S1 subunit of the Spike gene (though called “S2” primers), derived from Ref.^19^, and a spiked-in synthetic transcript of the same region that is added to each reaction at a fixed copy number for internal normalization across wells, which can be distinguished from the viral sequence on the basis of a 6 base pair (bp) barcode. After sequencing the amplified cDNA products, the readout of viral load is a log-transformed ratio of the number of viral RNA counts divided by the number of synthetic “spike-in” transcript counts (“S2/spike-in” ratio). After developing the assay, the creators released a publicly available protocol under a so-called “COVID license” and established a Users Group to facilitate implementation of this and other protocols.

Motivated in part by an apparent inadequate amount of testing in our own state of Arizona^20^, we participated in these meetings and began to evaluate the possibility of implementing Swab-Seq as a viable testing strategy in our community. We first evaluated the analytical sensitivity of the assay in our hands using contrived samples (i.e., samples to which synthetic viral RNA had been added), and then by leveraging samples collected as a part of ongoing studies at our campus testing facility, we explored the suitability of Swab-Seq for routine monitoring of asymptomatic populations by directly comparing Swab-Seq results with the results obtained using the traditional qRT-PCR assay on the same samples. In addition, we used Swab-Seq to test non-traditional sample types: saliva and saline gargle. We found that the assay was easy to implement, comparable to qRT-PCR in terms of clinical sensitivity in asymptomatic individuals (even in our non-CLIA setting), and that it is flexible to various sampling methods (including non-invasive and extraction-free sampling methods). Our results validate and extend recent method development^4,13–15^ and demonstrate the potential for more streamlined workflows for viral testing in the current pandemic and in the future.

## Results

### Swab-Seq is robust across various PCR kits and templates

**Figure 1:**
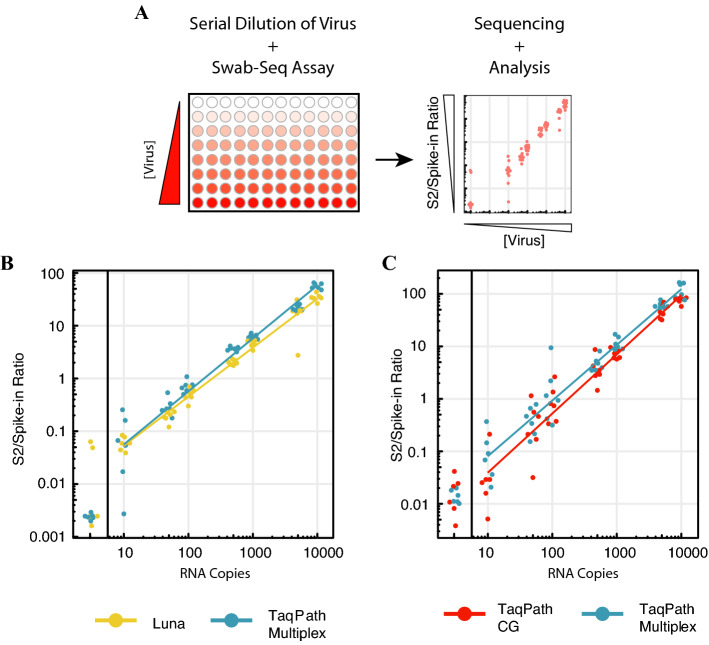
Implementation of Swab-Seq. (**A**) Schematic of initial QC experiments for implementing Swab-Seq. Synthetic RNA or inactivated virus is added to a 96-well plate at specified concentrations and then processed through the Swab-Seq assay. After sequencing the libraries, the S2/Spike-in ratio is compared to the loaded amount of viral material to determine the analytical sensitivity and robustness of the assay. (**B**) Comparison of Luna and TaqPath Multiplex 1-step RNA kits used in the assay. (**C**) Comparison of TaqPath Multiplex and TaqPath CG kits. In (**B**) and (**C**), black vertical lines partition 0 copies of virus from the serial dilutions. Each dot is an individual well of a plate with the specified concentration. The colored lines are least-squares fitted lines incorporating all non-zero data points.

To evaluate the implementation of Swab-Seq, we initially performed a set of quality control experiments (Fig. 1A). As in Ref.^14^, we used serial dilution of either synthetic viral RNA (Twist Biosciences) or heat-inactivated SARS-CoV-2 (ATCC) to establish the analytical sensitivity and linearity of the assay. With synthetic RNA, we estimated that the assay had a limit of detection (LOD) of approximately 10 copies of viral genome per reaction (equivalent to 2000 genomic copy equivalents or ‘GCE’ per mL of sample): 10 of 12 replicates were distinguishable from the negative controls at this viral load in our initial experiments with the AB TaqPath 1-step Multiplex Master Mix kit (blue dots in Fig. 1B,C, see also Supplementary Fig. S1, Supplementary Tables S1, S2), and an additional 12 of 12 replicates at this load were distinguishable from negatives regardless of whether we used synthetic RNA or inactivated viral particles in a subsequent experiment (Supplementary Fig. S2, Supplementary Table S3). While our estimates do not meet the FDA EUA threshold (requiring 19 of 20 replicates to be distinguishable at the LOD), we note that in two existing EUA approvals for Swab-Seq, the LOD was determined to be 125 GCE/mL^21^ and 250 GCE/mL^22^. It is worth also noting that in both cases the applicants spiked-in viral particles before RNA extraction, which would effectively concentrate viral RNA. Therefore, our estimate of ~ 2000 GCE/mL represents an estimate of the LOD without concentrating the sample. We also confirmed that the assay works well with three different 1-step qRT-PCR kits: NEB Luna and two different formulations of AB TaqPath (Fig. 1B,C, Supplementary Fig. S1, Supplementary Tables S1, S2), partially overlapping the kits used in Ref.^14^. In contrast to the Luna kit, the TaqPath kits contain Uracil-*N*-glycosylase (UNG), which helps to eliminate contamination from previously carried-over PCR products. Therefore, we used one of the TaqPath qRT-PCR kits, the TaqPath 1-step Multiplex Master Mix kit, for most experiments.

### Swab-Seq for community surveillance testing

**Figure 2:**
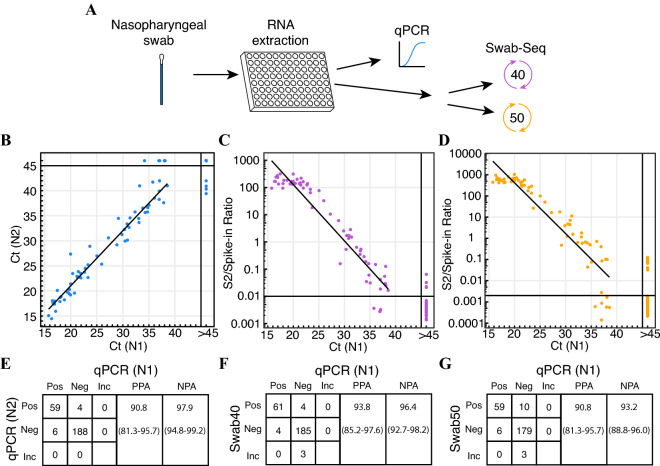
Evaluation of Swab-Seq as a community surveillance assay. (**A**) Schematic of testing regimen. After RNA extraction, all samples were tested via qRT-PCR, Swab-Seq with 40 cycles of amplification, or Swab-Seq with 50 cycles of amplification. (**B**) Ct values from qRT-PCR (average of triplicates) for N1 primer set (x-axis) are compared to Ct values from qRT-PCR (average of triplicates) for N2 primer set (y-axis). (**C**) Ct values (average of triplicates) for N1 primer set (x-axis) are compared to S2/Spike-in ratio for Swab-Seq with 40 cycles (y-axis). (**D**) Ct values (average of triplicates) for N1 primer set (x-axis) are compared to S2/Spike-in ratio for Swab-Seq with 50 cycles (y-axis). Black vertical lines partition “negative” from “positive” samples. Each dot is an individual sample. The diagonal lines are least-squares fitted lines incorporating all samples that are “positive” by both assays. (**E**–**G**) Tables of the number of samples categorized as positive or negative for the presence of the virus is presented between N1 qRT-PCR as the comparator assay and (**E**) N2 qRT-PCR, (**F**) Swab-Seq40, and (**G**) Swab-Seq50. Positive (“Pos”), negative (“Neg”), and inconclusive (“Inc”) results are compared pairwise. Positive % agreement (“PPA”) and negative % agreement (“NPA”) are provided for each comparison, along with 95% confidence intervals (in parentheses) calculated with the “Wilson” method from Ref.^25^.

Swab-Seq was previously demonstrated to discriminate between symptomatic patient samples and negative controls^14^ as stipulated in the FDA emergency use authorization (EUA) guidelines^21–23^. However, since testing regimens have become more commonplace for general surveillance programs, we were interested to see how the assay would perform relative to the gold standard qRT-PCR in a general population as well as in symptomatic individuals (Fig. 2A). To assess the feasibility of community surveillance with Swab-Seq, we tested four 96-well plates of samples that included 257 RNA samples extracted from NP swabs and 127 control wells (controls further explained in “Methods”). The samples represented on these plates were originally collected from two groups—a group of 81 symptomatic individuals and a group of 176 asymptomatic individuals. These samples (and the controls) had been tested previously with the standard qRT-PCR method using the CDC-recommended primers (for the N1 and N2 loci) under CLIA conditions.

Among the samples provided for our study, 60% (49/81) of symptomatic individuals and 9% (16/176) of asymptomatic individuals were qRT-PCR positive with the N1 primers. The Ct values for N1 and N2 were in high correlation (Pearson’s r = 0.97, p-value = 4.2 × 10^–38^, Fig. 2B) for all samples with detectable virus, although the two primer sets were not perfectly concordant in identifying positive samples at this Ct threshold (65 for N1, 63 for N2, 59 detected in common). Comparing the clinical sensitivity of the two qRT-PCR assays, the positive % agreement for N2 with N1 qRT-PCR was determined to be 91% (95% confidence interval (CI) 81.3–95.7%; Fig. 2E). The negative % agreement was 97.9% (95% CI 94.8–99.2%). Cohen’s kappa coefficient (κ)^24^, a metric for inter-rater reliability, for this comparison was 0.9 (95% CI 0.83–0.96).

Upon testing with Swab-Seq (Fig. 2, Supplementary Fig. S3, Supplementary Table S4), three subject samples (and one control sample) did not meet our threshold for sequencing depth (> 500 reads mapped to S2 and the spike-in control combined) and were considered inconclusive by Swab-Seq. We found that qRT-PCR Ct values for N1 and the Swab-Seq Log_10_-transformed S2/Spike-in ratios were highly correlated (Pearson’s r = − 0.96 for the 61 samples detectable by both methods, p-value = 1.0 × 10^–34^). The correlation was particularly evident in the range of ~ 20 < Ct <  ~ 40 (Fig. 2C), although Swab-Seq appears to show some saturation at very high viral load. Reassuringly, Ct values for *RPP30* (human control gene) were not correlated with the log-transformed Swab-Seq ratio (Pearson’s r = − 0.04, p-value = 0.5, Supplementary Fig. S3E). The correlation of *RPP30* levels from the Swab-Seq assay with the qRT-PCR Ct values of *RPP30* was initially low for samples as well (Pearson’s r = − 0.46, p-value = 1.0 × 10^–13^, Supplementary Fig. S3F). However, this rose substantially after we filtered out samples with such high viral loads that they suppressed the spike-in control counts that we utilized as a normalizing factor in the Swab-Seq method (Pearson’s r = 0.73, p-value = 3.2 × 10^–35^ after excluding 34 samples with < 600 spike-in control reads, Supplementary Fig. S3G). In addition, we observed no evidence of plate effects influencing the correlation between qRT-PCR and Swab-Seq (Supplementary Fig. S4). Importantly, Swab-Seq had a positive % agreement of 94% (61/65) with N1 qRT-PCR (95% CI 85.2–97.6%; Fig. 2F), higher than the N2 qRT-PCR primer set. The negative % agreement was 96.4% (95% CI 92.7–98.2%). The κ coefficient was 0.89 (95% CI 0.83–0.95). Swab-Seq also detected viral RNA in both of the S2-positive control wells. Among 124 negative control wells (including four that contain N control sequence but not S2), Swab-Seq gave a positive result for four, with three of the four having been positive in the N1 qRT-PCR assay as well, thus suggesting a false positive rate for Swab-Seq of 0.8% (1/121).

While this initial test was performed with 40 cycles for Swab-Seq (“Swab-Seq40” from here on), we noted that 50 cycles (“Swab-Seq50”) had been used in some instances in Ref.^14^, purportedly for better sensitivity. To test this directly, we re-processed all of the samples using Swab-Seq50 (Fig. 2A,D, Supplementary Fig. S5A, Supplementary Table S5). Three of the subject samples and four of the negative controls did not meet our threshold for combined S2 and spike-in reads (> 500 reads combined) this time as well. Comparing Swab-Seq50 and qRT-PCR, the positive % agreement was 90.8% (95% CI 81.3–95.7%; Fig. 2G), with a Pearson’s r = − 0.95 (p-value = 3.9 × 10^–31^) for samples positive by both assays (Fig. 2D). Interestingly, the negative % agreement decreased from 96.4% for Swab-Seq40 to 93.2% (95% CI 88.8–96.0%), which could indicate either that Swab-Seq50 is more able to identify true positives or that it is more prone to false positives. The κ coefficient was 0.81 (95% CI 0.73–0.89). Comparing the two Swab-Seq tests directly, the Pearson’s r was 0.98 (p-value = 5.2 × 10^–40^) for subject samples positive by both, and Swab-Seq50 detected 88% (57/65) of the samples detected as positive with Swab-Seq40. It was also apparent that the S2/Spike-in ratio values were lower for negative samples and higher for positive samples with Swab-Seq50 than with Swab-Seq40 (Supplementary Fig. S5B), indicative of a likely true gain in sensitivity. Swab-Seq40 identified eight positives that were not corroborated by Swab-Seq50 (four of which were also positive by N1 qRT-PCR), while Swab-Seq50 identified 12 that were not detected with Swab-Seq40 (two of which were also N1 qRT-PCR positive). The two positive controls containing S2 sequences were detected as positive by Swab-Seq50 (as they had been by Swab-Seq40). However, 11 of the negative control samples were detected as positive in the Swab-Seq50 assay (three of which were also detected by qRT-PCR), suggesting a potential false positive rate as high as 6.8% (8/118). We interpret these data to suggest that in some cases Swab-Seq50 missed true positives and included false positives, while the general trend seems to be that Swab-Seq50 overall is able to detect more positives (Fig. 2, Supplementary Fig. S5B).

### Swab-seq works in saliva

**Figure 3:**
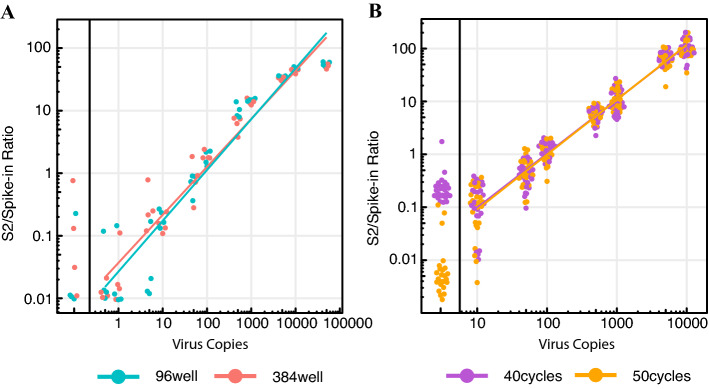
Swab-Seq in Saliva. (**A**) Comparison of 96-well and 384-well formats for testing saliva samples over a wide range of concentrations (0.5 to 100,000 copies per reaction). Points are colored by the plate format (96-well vs 384-sell). (**B**) Comparison of enzyme, denaturation time, and PCR cycle number in saliva. Points are colored by PCR cycle number here. For both (**A**) and (**B**), black vertical lines partition 0 copies of virus from the serial dilutions. Each dot is an individual well of a plate with the specified concentration. The colored lines are least-squares fitted lines incorporating all non-zero data points.

In the original Swab-Seq manuscript^14^, the authors demonstrated that Swab-Seq could be used to detect viral RNA directly (without extraction) from saliva samples following a protocol developed in Ref.^13^. To confirm that the saliva protocol worked in our hands, we spiked in heat-inactivated virus at specific concentrations to saliva collected from confirmed negative subjects, heated the samples to 95 °C for 30 min, diluted it 1:1 with a mixture of TBE and Tween-20, and loaded the samples directly into the 1-step PCR reactions. In this experiment, we included both 96- and 384-well plates to evaluate scalability. We found that the assay was sensitive down to ~ 10–50 copies/reaction (4000–20,000 copies/mL in saliva before dilution, Fig. 3A, Supplementary Fig. S6, Supplementary Table S6) in both formats. To further explore important variables in this assay, we tested three major comparisons in a single experiment: (1) the amount of time the samples were heated (the original publication of the saliva conditions indicated that 30 min at 95 °C was required for best qRT-PCR efficiency^13^), (2) the performance of both TaqPath enzymes in this more challenging scenario (i.e. in the presence of saliva), and (3) the number of cycles of amplification (in the original Swab-Seq manuscript, the authors used 50 cycles for direct-to-PCR samples from subjects). While the varying times of heat treatment and the two different enzyme formulations gave equivalent results in our hands (Supplementary Fig. S7, Supplementary Table S7), we observed a major improvement in the performance of negative controls for 50 cycles relative to 40 cycles (Fig. 3B).

### Swab-Seq works in saline gargle

While the saliva results are promising, there are challenges to working with saliva—including variable viscosity and variable volume obtained, as well as some anecdotal reticence on the part of individuals to the idea of spitting into a tube. This adds difficulty to designing scalable, automated solutions for sample handling (although the authors of the original Swab-Seq manuscript present one potential approach^14^). An alternative method recently developed and implemented at our university is the use of saline gargle. In this protocol subjects are given a 5 mL bolus of saline that they gargle in the back of their mouth for a short period of time before depositing the solution into a collection tube. This method reduces the issue of viscosity and helps to normalize the volume obtained from each individual, although at the cost of substantially diluting the virus. On the basis of the listed advantages, our university recently implemented saline gargle as a standard testing practice. To evaluate this collection method with Swab-Seq50, we first performed a serial dilution of inactivated virus, as we had done with other sample types. While the original saliva protocol tested a range of heat inactivation times and temperatures and found incubation of the sample at 95 °C for 30 min to be optimal for detection of the virus, the gargle protocol was initially implemented with a 65 °C for 30 min inactivation. To evaluate this in the context of Swab-Seq50, we tested 65 °C for 30 min and 95 °C for 10, 20, or 30 min in a single experiment (Supplementary Fig. S8, Supplementary Table S8). In contrast to the original report for saliva (assessed at 100 copies per reaction)^13^, 65 °C for 30 min appeared equivalent to 95 °C for 10, 20, or 30 min down to 100 copies per reaction. With concentrations at or below 50 copies per reaction, the data for samples at 95 °C suggest some potential degradation of detectable virus.

**Figure 4:**
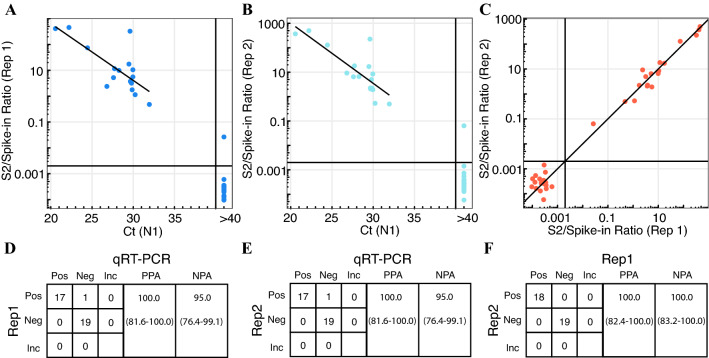
Swab-Seq and saline gargle for community surveillance. Saline gargle samples were obtained from 17 subjects with detectable virus (in qRT-PCR) and 20 samples with no detectable (qRT-PCR) virus. Samples were profiled in duplicate for Swab-Seq50. (**A**) Comparison of N1 qRT-PCR and S2/Spike-in ratio for replicate set 1. (**B**) Comparison of N1 qRT-PCR and S2/Spike-in ratio for replicate set 2. (**C**) Comparison of S2/Spike-in ratio for replicate set 1 with the ratio for replicate set 2. Dark vertical and horizontal lines denote the threshold for identifying positive samples in each assay (Ct < 40 for qRT-PCR, ratio > 0.002 for Swab-Seq50). The lines are least-squares fitted lines incorporating all “positive” data points for (**A**), (**B**). In (**C**), the line is the diagonal. (**D**–**F**) Tables of the number of samples categorized as positive or negative for the presence of the virus is presented between (**D**) replicate 1 or (**E**) replicate 2 and N1 qRT-PCR as the comparator assay, as well as (**F**) between replicate 2 and replicate 1 as the comparator. Positive (“Pos”), negative (“Neg”), and inconclusive (“Inc”) results are compared pairwise. Positive % agreement (“PPA”) and negative % agreement (“NPA”) are provided for each comparison, along with 95% confidence intervals (in parentheses) calculated with the “Wilson” method from Ref.^25^.

Since this gargle protocol was already implemented in our campus community for routine testing, we were also able to test gargle samples from some asymptomatic infected individuals. For this experiment, we tested 17 qRT-PCR-positive samples and 20 qRT-PCR-negative samples with Swab-Seq50 in duplicate. Comparing Ct values from the CDC-designed qRT-PCR assay to Swab-Seq50, we found that all 17 positive samples were detected with Swab-Seq50 in both replicate sets (Fig. 4A,B, Supplementary Table S9) and that one sample from the negative pool also appeared to be positive via Swab-Seq50 (Fig. 4C). Correlation for qRT-PCR with the Swab-seq50 positive samples yielded a Pearson’s r = − 0.75 (p-value = 5.4 × 10^–4^) and − 0.81 (p-value = 1.0 × 10^–4^) for replicate sets 1 and 2, respectively. The positive % agreement was 100% (95% CI 81.6–100.0%, Fig. 4D,E) between qRT-PCR and each Swab-Seq50 replicate, and the negative % agreement was 95% (95% CI 76.4–99.1%). The κ coefficient between both replicates and qRT-PCR was 0.95 (95% CI 0.84–1.00). Comparing Swab-seq50 replicates with each other, the Pearson’s r was 0.97 (p-value = 2.2 × 10^–12^), the positive % agreement was 100% (95% CI 82.4–100.0%, Fig. 4F), the negative % agreement was 100% (95% CI 83.2–100.0%), and the κ coefficient was 1.00 (95% CI: N/A). Although Swab-Seq only identified one additional positive sample, these results raise the possibility that Swab-Seq50 may be better able to detect positives than qRT-PCR for the routine surveillance of communities using a saline gargle protocol.

### SARS-CoV-2 in oral sampling methods is stable and compatible with Swab-seq

**Figure 5:**
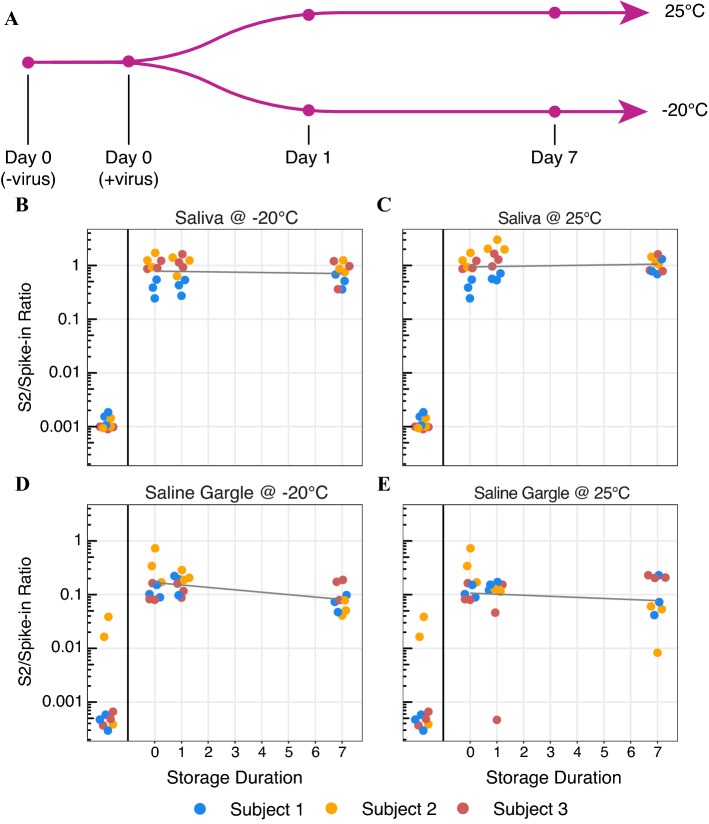
Stability of virus in saliva and saline gargle as assessed by Swab-Seq. (**A**) Schematic of virus stability study design. Saliva and saline gargle samples were collected from three individuals and inactivated virus was spiked-in such that the final PCR would contain 250 copies of the viral genome. The samples were then aliquoted and stored at 25 °C or − 20 °C for the indicated amount of time, at which point the sample was heated and stored. (**B**–**E**) Swab-Seq50 results for each time course: (**B**) saliva at − 20 °C, (**C**) saliva at 25 °C, (**D**) saline gargle at − 20 °C, and (**E**) saline gargle at 25 °C. Each sample was tested in triplicate. For each sample type (i.e. saliva and saline gargle), a single set of negative control samples and Day 0 samples were set up as reference for both storage conditions, so the data points for the negative and Day 0 controls are reproduced exactly in both panels. X-axis indicates storage duration in days, y-axis indicates S2/Spike-in ratio, and color of the point indicates the subject.

The fact that detectable SARS-CoV-2 viral RNA is reported to be very stable in saliva^26,27^ potentially allows for flexibility in the collection of samples for such an assay (including at-home collection and drop-off of samples). Therefore, we sought to confirm that Swab-Seq50 was able to detect virus in samples stored for extended periods at − 20 °C or at room temperature (Fig. 5A). These temperatures were chosen to represent the extremes at which diagnostic labs might consider storing samples. To test stability of the (heat-inactivated) virus, a time course experiment was set up by collecting saliva and saline gargle samples from three (virus-negative) subjects. We then spiked in heat-inactivated virus at a concentration 100 copies per μL for saliva and 50 copies per μL for saline gargle (the difference in concentration was to account for the subsequent TBE/Tween-20 dilution step in the saliva protocol). Samples were stored at either − 20 °C or at room temperature for up to one week. The samples were then heated for 30 min (95 °C for saliva, 65 °C for saline gargle), saliva samples (not gargle samples) were diluted with TBE plus Tween-20, all samples were frozen, and then all were assayed as a complete set at the end of the time course. Consistent with previous reports in saliva^15,27^, the virus appeared to be highly stable at both storage temperatures for either sample collection type (Fig. 5B–E, Supplementary Table S10). In order to evaluate this relationship statistically, we used a likelihood-ratio testing framework (see “Methods” for details) to determine if storage duration had a significant impact on viral abundance in any of the four conditions considered here. We did not find a significant effect for any of the conditions over the 7-day time course (p-values: saliva at − 20 °C = 0.52; saliva at RT = 0.99; gargle at − 20 °C = 0.09; gargle at RT = 0.65). However, saline gargle samples exhibited a compressed S2/Spike-in ratio (which we interpret as indicative of PCR inhibition). Nonetheless, viral particles were stably detectable across individuals and storage conditions.

## Discussion

In this study we evaluated the sensitivity, scalability, and flexibility of Swab-Seq as a potential assay for community surveillance of SARS-CoV-2. We found that the analytical sensitivity of the assay was ~ 10 copies per reaction in our research setting, and (consistent with previous results^14,21,22^) is likely even more sensitive in a CLIA setting. The assay is compatible with several enzyme kits and sample types, including RNA extracted from swabs and (unextracted) saliva and saline gargle samples. Saliva and saline gargle sample collection methods are particularly promising, as they require relatively little equipment or expertise for collection, are stable for at least a week at room temperature, and can be used as templates with minimal pre-processing. In our testing, saliva appears to have a lower limit of detection than the gargle, likely due to both the dilution factor and PCR inhibition effects of the saline solution used in the gargle. However, one limitation of our study is that all of our tests involving saliva were conducted with spiked-in viral particles, unlike our experiments with swabs and saline gargle for which samples from study subjects were available to evaluate clinical sensitivity and specificity. Future work will be required to compare saliva and saline gargle samples directly to NP swab samples from the same subjects.

There are several caveats to these results. First, while Swab-Seq50 does appear in general to be more analytically sensitive than Swab-Seq40 in head-to-head comparisons, it also appears to be susceptible to both false negatives and false positives (predominantly among samples near the limit of detection). We suspect this could be explained by PCR amplification biases^28^, though we have not evaluated this directly. Second, while the assay is fairly streamlined—implementing extraction-free sample collection methods, and sequencing as short as possible—this method does not represent a rapid diagnostic. All things considered, we expect that it is possible to process samples at approximately the same rate as the qRT-PCR standard in a diagnostic lab setting. Swab-Seq has the advantage that it can be scaled with more barcoded primers and more standard thermal cyclers, whereas the qRT-PCR assay requires additional (expensive) real-time thermal cyclers. However, Swab-Seq also requires access to an Illumina sequencer. In addition, as detailed in previous publications^14,29^, Swab-Seq is susceptible to a phenomenon called “barcode swapping”, in which residual primers are incorporated into PCR products on the sequencer after pooling samples and thereby can swap out the correct barcode for an erroneous one. In the experiments where we used unique dual indices (UDI), and were therefore best powered to detect barcode swapping, the percentage of reads that matched individual barcodes but represented invalid pairs of barcodes ranged from 0.03% on a NextSeq sequencer to 2% on a MiSeq sequencer (Supplementary Table S11). While the NextSeq data showed a lower swapping rate, these samples were also pooled with other libraries, which serves to both dilute the concentration of carried over primers and dilute the concentration of target amplicons from the same library (relative to amplicons from other libraries). Finally, we have emphasized the use of extraction-free methods for processing samples. These have important advantages, including limited handling of potentially infectious samples, long-term stability at room temperature, speed in processing, and scalability. However, extracting RNA both concentrates the sample and removes background contaminants that can inhibit PCR. We observe a particular reduction in the limit of detection in saline gargle relative to saliva and extracted RNA, even though this sample type is the most amenable to scaled processing. Nonetheless, in our view the advantages of Swab-Seq warrant its use in facilitating a response to the current pandemic and future ones.

Looking forward, it is our position that Swab-Seq (and assays like it) would benefit from further development of several modifications for even greater applicability and precision. First, multiplexing the assay would potentially improve the sensitivity and enable simultaneous testing for multiple viruses. This would help to both combat the SARS-CoV-2 pandemic and to enable more routine testing for seasonal viruses. Second, the issues with barcode swapping present a major challenge. Proposed solutions include the use of UDI where every well sequenced together receives a unique index on each end of the amplicon. This makes the identification of barcode swapping unambiguous, but does not scale well—profiling 384 samples requires 1536 unique primers. A second option is to use so-called “semi-combinatorial” indexing, whereby not all combinations of primers are implemented. Instead, several sets of independent primers may be used in a combinatorial fashion (e.g. one set used for each quadrant of a plate). This increases the overall diversity of barcodes relative to combinatorial indexing and also increases the number of combinations that are invalid, allowing for better exclusion of some swapping events. However, this leaves some barcode swapping undetectable and still has issues with scaling—splitting 384 into four groups that each received independent combinations would still require 160 primers. A third solution would be to introduce a unique molecular index (UMI) into the library design. This degenerate sequence would be introduced during reverse transcription and would uniquely tag each RNA molecule profiled. Through PCR amplification, proper UMIs would be copied in large quantities. Barcode swapping occurring later on the sequencing instrument, would result in a rare pairing of a UMI and a barcode combination that should be easy to detect and exclude. There are challenges in implementing a UMI in the current 1-step qRT-PCR workflow, but we think this would be an important future improvement. In addition, if the library construction design incorporated a third level of barcoding, 384 samples could reasonably be profiled by 48 primers in one configuration.

In our evaluation of Swab-Seq for surveillance testing, we found the implementation of the assay to be straightforward and flexible. Being at least as clinically sensitive as the current gold standard assay and presenting potential advantages in terms of scalability, flexibility regarding sample type, and cost, the existing techniques should allow for rapid, economical adaptation of this testing framework to a healthcare setting. While there are some limitations to the assay in its current design, it is amenable to further improvements that could allow for even more precise, flexible, and scalable testing in the future.

## Methods

### Synthesis of S2 spike-in

The S2 spike-in was synthesized as described in Ref.^14^. Briefly, S2 template cDNA with a T7 promoter and a barcode for in vitro transcription was obtained by amplifying 1 × 10^6^ copies of the synthetic SARS-CoV-2 RNA (Twist Bioscience) using the Luna Universal One-Step RT-qPCR kit (NEB, #E3005S) with 400 nM of forward and reverse primers (primer sequences in Supplementary Table S12). Cycling conditions were as follows: 55 °C for 10 min, 95 °C for 1 min, followed by 29 cycles of 95 °C for 10 s and 60 °C for 30 s. The synthesized cDNA was split into two equal aliquots and cleaned using either the Zymo DNA Clean & Concentrator-5 kit (Zymo Research, #D4013) or 2X AMPure XP beads. As there were no apparent differences running the two fractions on a gel, the aliquots were combined for downstream synthesis. 500 ng of template DNA was subjected to in vitro transcription using the NEB HiScribe T7 kit (NEB, #0204S) following the manufacturer's instructions. The reaction mixture was incubated for 16 h at 37 °C. The synthetic S2 spike-in was purified using RNA Clean & Concentrator-5 kit (Zymo Research, #ZR1013) according to the manufacturer's instructions and eluted with nuclease-free water. The synthesized spike-in RNA was quantified via Qubit, and the size of the RNA was verified by running it alongside a control S2 spike-in aliquot (kindly provided by Octant Bio) on a 6% Novex TBE gel (Invitrogen).

### Setting up dilution experiments

Serial dilutions of synthetic SARS-CoV-2 RNA (Twist Bioscience, #102019) or heat-inactivated SARS-CoV-2 virus (ATCC, #VR-1986HK) and *RPP30* DNA (IDT, #10006626) were freshly prepared for every experiment. Serial dilutions were done once for each experiment, and replicates represent additional aliquots of the same dilution. All dilutions were done by the same individual. For the comparison of TaqPath 1-step Multiplex Master Mix (Thermofisher, #A28521) with Luna Universal One-Step RT-qPCR kit (NEB, #E3005S), the mixture of diluted synthetic viral RNA and 2,000 copies of *RPP30* DNA per reaction was prepared with lysis buffer included in Cells-to-cDNA II kit (Invitrogen, #AM1722) and then heated at 75 °C for 10 min as described in Ref.^14^. For the dilution experiment comparing the TaqPath 1-step Multiplex Master Mix with the TaqPath CG (Thermofisher, #15299), the mixture of synthetic viral RNA and *RPP30* DNA was prepared with nuclease-free water without heating. For the dilution experiment comparing the viral RNA and viral particles, the serial dilutions were prepared in nuclease-free water without heating. See Supplementary Table S12 for sequences of primers used in these experiments.

### Extracted RNA from subjects for clinical diagnostics

RNA samples were collected from subjects as a part of routine surveillance testing on the University of Arizona campus. NP swab samples were collected from the upper respiratory system of each participant under the supervision of a health care professional and placed into 3 mL of the CDC formulation Viral Transport Media (VTM, https://www.cdc.gov/csels/dls/locs/2020/new_sop_for_creating_vtm.html). Swabs in VTM were stored at 4 °C until RNA extraction, which was performed within 72 h of collection for all samples. For testing, 200 μL of VTM was used for RNA extraction using Zymo *Quick*-RNA 96 Viral kit (Zymo Research) and eluted in 30 μL. All samples were stored in 96-well plates at − 20 °C and tested with the SARS-CoV-2 qRT-PCR assay within 24 h of isolation. All sample remnants were stored long term at − 80 °C after qRT-PCR testing. All samples were collected via physician blanket order. All participants in this study and the samples described below provided informed consent and this study was approved by both the University of Arizona COVID-19 Data Governance Committee and the Institutional Review Board. All methods were performed in accordance with the relevant guidelines and regulations.

### qRT-PCR of RNA extracted from NP swabs from subjects

The SARS-CoV-2 qRT-PCR testing procedure used is a modification of the CDC recommended protocol (https://www.cdc.gov/coronavirus/2019-ncov/lab/virus-requests.html). The assay implements the primer/probe pairs provided in the 2019-nCoVEUA-01 Diagnostic Panel Primer set [Integrated DNA Technologies (IDT)], including the human *RNaseP* gene (referred to here as “*RPP30*”) as a functional RNA isolation control marker. Additionally, the 2019-nCoV_N_Positive Control (IDT) was run in tandem on each 96-well plate of clinical sample testing. A one-step qRT-PCR reaction was performed using 5 μL of sample run individually for each of the three regions (*N1*, *N2* and *RPP30*) in a total reaction volume of 10 μL using the TaqPath™ 1-Step RT-qPCR CG Master Mix (Thermofisher, #A15300). All testing was performed using the ABI PRISM^®^ 7900HT Sequence Detection System (Applied Biosystems) and all sample processing runs contained at least one positive, negative and no template control. A cut off of raw cycle threshold (Ct) values < 45 was used to determine positivity for all 3 regions in this study.

The 4 plates of samples used in these analyses included 257 samples collected from two sources of asymptomatic or symptomatic individuals (indicated by sample IDs starting with “ASYMPT” or “SYMPT”), 125 negative control wells (indicated in the sample ID by terms “NEG” for wells just containing water, “EXT” for wells containing RNA extracted from uninfected cells, or “NPOS” for the IDT 2019-nCoV_N_Positive Control *N* gene control) and 2 positive control samples (“SPOS”) created from pools of previously positive samples.

### Preparing saliva samples for dilution/stability experiments

For dilution/stability experiments, subjects negative for COVID-19 provided saliva samples by spitting into 5 mL of saliva into a sterile 50 mL conical tube. Saliva samples were vortexed before transferring 100 μL into 1.5 mL tubes and mixed with a given concentration of serially diluted, heat-inactivated virus thoroughly by vortexing and pipetting up and down. Saliva samples with viral particles spiked in were heated at 95 °C for 10–30 min, depending on the experiment, diluted 1:1 with 100 μL of 2X TBE + 1% Tween-20, and then placed on ice until the next step.

### Preparing saline gargle samples for dilution/stability experiments

For dilution/stability experiments, subjects negative for COVID-19 provided gargle samples, by tilting their heads back and gargling a 5 mL 0.9% NaCl saline bolus (AddiPak cat# 200-59) for a minimum of 30 s, before spitting the saline into a collection tube. As with the saliva samples, gargle samples were vortexed before transferring 200 μL into 1.5 mL tubes and mixed with a given concentration of serially diluted, heat-inactivated virus thoroughly by vortexing and pipetting up and down. Gargle samples with viral particles spiked in were heated at 65 °C for 30 min or 95 °C for 10–30 min, depending on the experiment, and then placed on ice until the next step.

### Gargle samples from subjects for clinical diagnostics

Saline gargle samples were collected from subjects as a part of routine surveillance on the University campus. Subjects were provided with a 5 mL bolus of sterile saline 0.9% NaCl (AddiPak cat# 200-59) and a sterile collection tube and instructed to gargle the saline with their head tilted back for 30 s followed by a 5 s swish, and repeated 3 times before depositing the solution in the collection tube and capping the sample. Samples were stored immediately at 4 °C. Upon transfer to the diagnostic lab, samples were incubated at 65 °C for 30 min and then stored at − 80 °C until ready for testing. Samples were collected under physician blanket order.

### qRT-PCR of saline gargle samples from subjects

All samples were tested in triplicate at a volume of 10 μL using the CDC *N1* and *RPP30* primer/probe sets using the identical qRT-PCR reagents and conditions described for NP swabs, except that samples were amplified for 40 cycles instead of 45.

### Swab-Seq PCR reactions

Swab-Seq reactions were conducted with either the Luna one-step universal RT master mix or one of two TaqPath master mixes (Multiplex or CG) in a 20 μL reaction volume using a C1000 Touch (Bio-Rad) thermocycler. The one-step master mix containing 500 copies of S2 spike-in per reaction and Luna or TaqPath reagents was dispensed into each well of a PCR plate. Next, the primer mixture of barcoded S2 (400 nM final concentration for both primers) and *RPP30* (50 nM final concentration for both) primers was added to each well. 5 μL of template were added to the corresponding wells as the last step before running PCR. Plates were sealed with Optical seals (Bio-Rad #MSB1001). The cycling profiles for each RT-PCR kit are as follows:

- Luna kit: 55 °C for 10 min, 95 °C for 1 min, 40 cycles of 95 °C for 10 s and 60 °C for 30 s.
- TaqPath multiplex: 25 °C for 2 min, 53 °C for 10 min, 95 °C for 2 min, 40 or 50 cycles of 95 °C for 10 s and 60 °C for 30 s.
- TaqPath CG: 25 °C for 2 min, 50 °C for 15 min, 95 °C for 2 min, 40 or 50 cycles of 95 °C for 3 s and 60 °C for 30 s.

### qRT-PCR of other samples

To confirm subjects were negative for dilution/stability experiments, some samples were subjected to qRT-PCR. The qRT-PCR for these samples was conducted with TaqPath 1-step Multiplex Master Mix and *N1* Taqman probes (IDT #10006713) in a 20 μL of reaction volume using the CFX Connect Real-Time PCR System (Bio-Rad). 5 μL of template was loaded per reaction. The thermocycling profile is the same as described above for Swab-Seq.

### Purification and preparation of sequencing library

Swab-Seq PCR products were pooled by transferring 5 μL of each well into a new 1.5 mL tube and then cleaned up with 2X AMPure XP beads (Beckman Coulter, #A63881). One volume of pooled samples and two volumes of beads were mixed and incubated for 5 min at room temperature, followed by placing the sample tubes on a magnetic stand for ~ 2 min to separate the beads and solution. The supernatant was then discarded. After aspiration, the beads were washed twice with 200 μL of 80% ethanol on a magnetic stand, dried at room temperature for 5 min, and then removed from the magnetic stand and resuspended in 20 μL of EB buffer (Qiagen, #19086). After incubating for 2 min, the sample was placed back on the magnetic stand to separate the beads for an additional ~ 2 min. The supernatant was then transferred into a new tube. The concentration of eluted DNA was determined using the Qubit 1× dsDNA HS assay kit (Invitrogen, #Q33231). While Swab-Seq libraries were found to produce variable band sizes across libraries, the size of the major band (200 bp), representing the S2 PCR product, was verified by running a 6% Novex TBE gel (Supplementary Fig. S9). We assumed an average library size of 200 bp when converting the ng/μL concentration into a molar concentration using the following equation: (concentration in ng/μL)/(660 g/mol × average library size in bp) × 10^6^ = concentration in nM.

### Gel imaging equipment and settings

For gel imaging of PCR products, 1 μL of each product was loaded on a 1× TBE gel along with 1× Orange Gel Loading Dye (NEB, #B7022S) and run at 180 V for 30 min. 50 ng of a 100 bp DNA ladder (NEB, #N3231S) were also run alongside samples as a standard. After running, gels were stained with 1× SYBR Gold (Invitrogen, #S11494) in 1× TBE buffer for approximately 1 min and imaged via blue light on an Axygen Gel Documentation System-BL. For Supplementary Fig. S9, the gel was exposed to blue light for 1 s and 143 ms.

### Sequencing

For sequencing, the purified library was freshly diluted to 2 nM with EB buffer, and then 10 μL of the library was denatured with 10 μL of 0.1 N NaOH by incubating for 5 min at room temperature. The addition of 980 μL of HT1 buffer (Illumina) stopped the denaturation and diluted the library to 20 pM. The PhiX (Illumina) control (when Swab-Seq was not pooled with other sample types) was also prepared at 20 pM in the same way, and both the denatured library (60%) and the PhiX control (40%) were mixed in these instances. Libraries were then further diluted to 8 pM (MiSeq v2 kit), 15 pM (MiSeq v3 kit), or 1.5 pM (NextSeq Mid kit) with HT1 buffer.

Custom primers for Read1 and Index1(also called “i7”) (and Index2 (also called “i5”), if sequencing on a NextSeq) were spiked into the standard primer reservoirs. For the MiSeq, 100 μL of the primer mixture in reservoir 12 was mixed with 6.8 μL of the custom Read1 primer mixture (50 µM of each primer for S2 and *RPP30*) and then replaced in reservoir 12 of the MiSeq reagent cartridge. The i7 primer mixture for S2 and *RPP30* was loaded into reservoir 13 with the same method. For the NextSeq 500/550, 52 μL of the Read1 primer mixture (each primer at a final concentration of 10 uM) was loaded into reservoir 22. In addition, 85 μL of the i7 primer mixture (10 μM for each primer) and 85 μL of the i5 primer mixture (10 µM for each primer) were loaded into reservoir 23. Unless accommodating for other library types on the same run, sequencers were run with 26 cycles of read 1 and 10 cycles of each index. See Supplementary Table S11 for a summary of the sequencing results. Note that we re-sequenced one sample, “Saliva_dilutions_96_vs_384”, three times due to low clustering on the sequencer. Here we have combined all three runs for analysis.

### Analysis

BCL files from the sequencers were converted to fastq files locally on a personal computer using Illumina’s bcl2fastq program implemented as a Docker image from (https://hub.docker.com/r/genomicpariscentre/bcl2fastq2). Because some samples were multiplexed with other experiments, we developed a custom python script to demultiplex fastq files on the basis of expected barcodes while accounting for other barcodes that were sequenced on the same run and allowing for a hamming distance of 1 between sequenced barcodes and expected/possible barcodes. For consistency, even experiments sequenced alone were demultiplexed with this pipeline. The demultiplexing script is available from our github repository (https://github.com/cusanovichlab/swabseq). Plate maps were generated and then converted into sample sheets using the code available from Octant Bio’s github repository (https://github.com/octantbio/SwabSeq). Metadata files and whitelists of barcodes were generated using a previously published preprocessing method^29^ (outlined in a Jupyter notebook for Swab-Seq analysis https://github.com/pachterlab/BLCSBGLKP_2020/blob/master/notebooks/swabseq.ipynb). Fastq files were then mapped to a custom reference that included the S2, spike-in control, and *RPP30* sequences and summed by sample barcode using the “Swabseq10” command available on the “covid” branch of the kallisto Github repository (https://github.com/pachterlab/kallisto.git) as well as bustools^30,31^. We implemented these steps in a Docker container locally as well.

After generating a data matrix that provides read counts for each transcript assigned to each sample barcode, we calculated S2/Spike-in ratios and visualized data in R by modifying scripts from the Octant Github repository. We provide our scripts for data processing and visualization as a Github repository (https://github.com/cusanovichlab/swabseq). For samples collected from asymptomatic individuals (both extracted RNA and saline gargle), we obtained Ct values as the average of three qRT-PCR replicates from the parent study. We considered any sample with a detectable Ct value positive. For Swab-Seq40, we set the S2/Spike-in ratio positive samples at 0.01. For Swab-Seq50, we set the threshold at 0.002. All titration and stability experiments were conducted with combinatorial barcodes, such that each well of each plate received a unique combination of i5 and i7 barcodes and all samples were included in downstream analyses regardless of sequencing depth. All experiments involving subject samples were conducted with UDI, meaning that every well received a unique i5 barcode and a unique i7 barcode. These UDI primers were kindly provided by Octant Bio. Only samples with at least 500 reads mapping to either S2 or the spike-in control were considered in downstream analyses for experiments with subject samples. While Ct values represented the average of triplicates, S2/Spike-in ratio values were determined without replicates.

### Comparisons of diagnostic tests

Comparisons between diagnostic tests were assessed with both positive/negative percent agreement and Cohen’s kappa coefficient^24^. 95% confidence intervals on positive percent agreement and negative percent agreement metrics were calculated using the “Wilson” method from Ref.^25^. Cohen’s kappa coefficient and 95% confidence intervals were calculated using the “cohen.kappa” function from the “psych” package^32^ in R. For both metrics, inconclusive samples were included as a third category.

### Likelihood-ratio testing framework for viral stability

In order to statistically evaluate the stability of viral particles stored in various conditions, we adopted a likelihood-ratio testing framework using fixed-effect linear models. Namely, we fit a linear model to the data such that Y = μ + βX + αZ + ε, where Y represents the Swab-Seq estimates of viral abundance (which are assumed to be normally distributed), “μ” represents the mean abundance, “X" represents the duration of storage (0, 1, or 7 days as a numerical value), “β” represents the effect of storage duration, “Z” represents the individual tested (since there were three subjects included in the study), “α” represents the effect of individuals on the mean viral abundance, and “ε” represents the residual error. The nested model did not include a term for storage duration (i.e. Y = μ + αZ + ε). Log likelihoods were calculated with the ‘logLik’ and ‘lm’ functions in R, and p-values were calculated from the test statistics based on a χ^2^ distribution with two degrees of freedom.

## Supplementary Information

Supplementary Figures.

Supplementary Table S1.

Supplementary Table S2.

Supplementary Table S3.

Supplementary Table S4.

Supplementary Table S5.

Supplementary Table S6.

Supplementary Table S7.

Supplementary Table S8.

Supplementary Table S9.

Supplementary Table S10.

Supplementary Table S11.

Supplementary Table S12.

## Data Availability

Data generated for this study are available from Gene Expression Omnibus (GSE176224).

## References

[1] ProMED-mail. Undiagnosed Pneumonia—China (Hubei): Request for Information. ProMED-mail 2019. 20191230.6864153 (2019).

[2] Wu F, Zhao S, Yu B, Chen Y-M, Wang W, Song Z-G (2020). A new coronavirus associated with human respiratory disease in China. Nature.

[3] Diagnostic detection of Wuhan coronavirus 2019 by real-time RTPCR. https://www.who.int/docs/default-source/coronaviruse/wuhan-virus-assay-v1991527e5122341d99287a1b17c111902.pdf?sfvrsn=d381fc88_2. Accessed 15 May 2021.

[4] Srivatsan S, Heidl S, Pfau B, Martin BK, Han PD, Zhong W (2020). SwabExpress: An end-to-end protocol for extraction-free COVID-19 testing. bioRxiv.

[5] Wang W, Xu Y, Gao R, Lu R, Han K, Wu G (2020). Detection of SARS-CoV-2 in different types of clinical specimens. JAMA.

[6] McFee DRB (2020). COVID-19 laboratory testing/CDC guidelines. Dis Mon..

[7] World Health Organization. Laboratory testing for coronavirus disease 2019 (COVID-19) in suspected human cases: Interim guidance, 2 March 2020. World Health Organization; 2020. Report No.: WHO/COVID-19/laboratory/2020.4. https://apps.who.int/iris/handle/10665/331329. Accessed 15 May 2021.

[8] Koskinen A, Tolvi M, Jauhiainen M, Kekäläinen E, Laulajainen-Hongisto A, Lamminmäki S (2021). Complications of COVID-19 nasopharyngeal swab test. JAMA Otolaryngol. Head Neck Surg..

[9] Palmas G, Moriondo M, Trapani S, Ricci S, Calistri E, Pisano L (2020). Nasal swab as preferred clinical specimen for COVID-19 testing in children. Pediatr. Infect. Dis. J..

[10] Callahan C, Lee RA, Lee GR, Zulauf K, Kirby JE, Arnaout R (2020). Nasal-swab testing misses patients with low SARS-CoV-2 viral loads. medRxiv.

[11] Wyllie AL, Fournier J, Casanovas-Massana A, Campbell M, Tokuyama M, Vijayakumar P (2020). Saliva or nasopharyngeal swab specimens for detection of SARS-CoV-2. N. Engl. J. Med..

[12] Moore C, Corden S, Sinha J, Jones R (2008). Dry cotton or flocked respiratory swabs as a simple collection technique for the molecular detection of respiratory viruses using real-time NASBA. J. Virol. Methods..

[13] Ranoa DRE, Holland RL, Alnaji FG, Green KJ, Wang L, Brooke CB (2020). Saliva-based molecular testing for SARS-CoV-2 that bypasses RNA extraction. bioRxiv.

[14] Bloom JS, Sathe L, Munugala C, Jones EM, Gasperini M, Lubock NB, *et al.* Swab-Seq: A high-throughput platform for massively scaled up SARS-CoV-2 testing. *medRxiv*. 2020.08.04.20167874 (2021).

[15] Vogels CBF, Watkins AE, Harden CA, Brackney DE, Shafer J, Wang J (2021). SalivaDirect: A simplified and flexible platform to enhance SARS-CoV-2 testing capacity. Med (N Y)..

[16] David Paltiel A, Zheng A, Walensky RP (2020). Assessment of SARS-CoV-2 screening strategies to permit the safe reopening of college campuses in the United States. JAMA Netw Open..

[17] Hellewell, J., Russell, T. W., The SAFER Investigators and Field Study Team, The Crick COVID-19 Consortium, CMMID COVID-19 working group, Beale, R., *et al.* Estimating the effectiveness of routine asymptomatic PCR testing at different frequencies for the detection of SARS-CoV-2 infections. *medRxiv*. 2020.11.24.20229948 (2020). 10.1186/s12916-021-01982-x PMC8075718 33902581

[18] Larremore DB, Wilder B, Lester E, Shehata S, Burke JM, Hay JA (2020). Test sensitivity is secondary to frequency and turnaround time for COVID-19 surveillance. medRxiv.

[19] Won J, Lee S, Park M, Kim TY, Park MG, Choi BY (2020). Development of a laboratory-safe and low-cost detection protocol for SARS-CoV-2 of the coronavirus disease 2019 (COVID-19). Exp. Neurobiol..

[20] Kliff, S. Arizona “Overwhelmed” with demand for tests as U.S. system shows strain. The New York Times. 25 Jun 2020. https://www.nytimes.com/2020/06/25/upshot/virus-testing-shortfall-arizona.html. (accessed 29 May 2021).

[21] U.S. Food and Drug Administration. Emergency Use Authorization (EUA) Summary for Helix COVID19 NGS Test. https://www.fda.gov/media/140917/download. Accesssed 13 Jan 2022.

[22] U.S. Food and Drug Administration. Emergency Use Authorization Summary UCLA SwabSeq COVID-19 Diagnostic Platform. https://www.fda.gov/media/142805/download. Accesssed 13 Jan 2022.

[23] Center for Devices, Radiological Health. Policy for COVID-19 Tests During the Public Health Emergency (Revised). 2020. https://www.fda.gov/regulatory-information/search-fda-guidance-documents/policy-coronavirus-disease-2019-tests-during-public-health-emergency-revised. Accesssed 29 May 2021.

[24] Cohen J (1960). A coefficient of agreement for nominal scales. Educ. Psychol. Meas..

[25] Altman D (2000). Statistics with Confidence: Confidence Intervals and Statistical Guidelines.

[26] Griesemer SB, Van Slyke G, Ehrbar D, Strle K, Yildirim T, Centurioni DA (2021). Evaluation of specimen types and saliva stabilization solutions for SARS-CoV-2 testing. J. Clin. Microbiol..

[27] Ott IM, Strine MS, Watkins AE, Boot M, Kalinich CC, Harden CA (2020). Simply saliva: Stability of SARS-CoV-2 detection negates the need for expensive collection devices. medRxiv.

[28] Fu Y, Wu P-H, Beane T, Zamore PD, Weng Z (2018). Elimination of PCR duplicates in RNA-seq and small RNA-seq using unique molecular identifiers. BMC Genom..

[29] Sina Booeshaghi A, Lubock NB, Cooper AR, Simpkins SW, Bloom JS, Gehring J (2020). Reliable and accurate diagnostics from highly multiplexed sequencing assays. Sci. Rep..

[30] Bray NL, Pimentel H, Melsted P, Pachter L (2016). Near-optimal probabilistic RNA-seq quantification. Nat. Biotechnol..

[31] Melsted P, Sina Booeshaghi A, Gao F, Beltrame E, Lu L, Hjorleifsson KE (2019). Modular and efficient pre-processing of single-cell RNA-seq. bioRxiv.

[32] Revelle, W. Procedures for Psychological, Psychometric, and Personality Research [R package psych version 2.1.9]. 2021 https://CRAN.R-project.org/package=psych. Accesssed 13 Jan 2022.

