# Supplementary material for: Evaluation of Swab-Seq as a scalable, sensitive assay for community surveillance of SARS-CoV-2 infection
Source: Sci Rep. 2022 Feb 23;12:3047. doi: 10.1038/s41598-022-06901-5 (PMC8866503; doi:10.1038/s41598-022-06901-5)
Supplement: Supplementary file 1 — Supplementary Figures. [file 41598_2022_6901_MOESM1_ESM.pdf]

Supplementary Information

Evaluation of Swab-Seq as a scalable, sensitive assay for community surveillance of SARS-CoV-2 Infection

HyunJin Kang[1], Sheilah Allison[1], Amber Spangenberg[1], Tara Carr[1], Ryan Sprissler[2,3], Marilyn Halonen[1], Darren A. Cusanovich[1,4,5*]

1. Asthma and Airway Disease Research Center (A^{2}DRC), University of Arizona, Tucson, AZ
2. Center for Applied Genetics and Genomic Medicine, University of Arizona, Tucson, AZ
3. University of Arizona Genetics Core, University of Arizona, Tucson, AZ
4. Department of Cellular and Molecular Medicine, University of Arizona, Tucson, AZ
5. BIO5 Institute, University of Arizona, Tucson, AZ

*Correspondence: darrenc@arizona.edu

Supplementary Figures

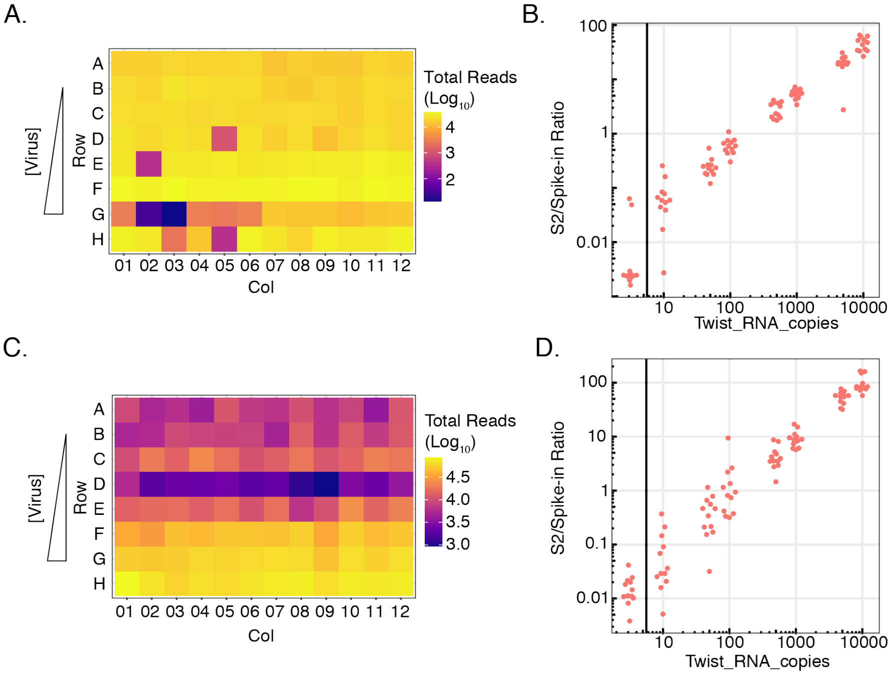

Figure S1. (A) Heatmap of $\log_{10}$(total read depth) for all samples for the serial dilution comparing Luna and TaqPath Multiplex 1-step qRT-PCR kits. Rows represent increasing concentrations of virus, from 0 copies in row A to 10,000 copies in row H. (B) Data points for number of loaded GCE of viral RNA (x-axis) and *S2*/Spike-in ratio (y-axis) without superimposed linear trends for the same comparison. (C) Heatmap of read depth for every sample for the serial dilution comparing TaqPath Multiplex and TaqPath CG 1-step qRT-PCR kits. Rows represent increasing concentrations of virus, from 0 copies in row A to 10,000 copies in row H. (D) Data points for number of loaded GCE of viral RNA (x-axis) and *S2*/Spike-in ratio (y-axis) without superimposed linear trends for the same comparison.

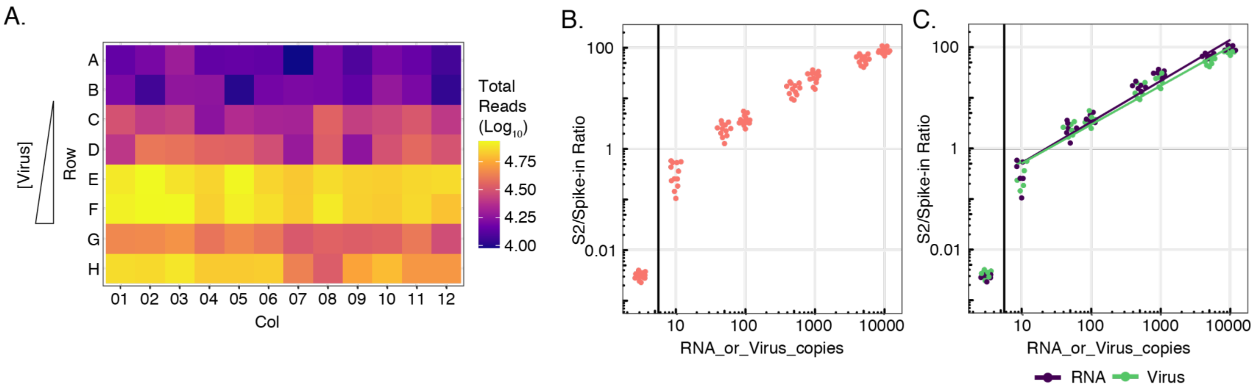

Figure S2. (A) Heatmap of $\log_{10}$(total read depth) for all samples for the serial dilution comparing synthetic RNA template and heat-inactivated virus template. Rows represent increasing concentrations of viral load, from 0 copies per reaction in row A to 10,000 copies in row H. (B) Scatterplot for the number of loaded GCE of viral RNA or particles (x-axis) and *S2*/Spike-in ratio (y-axis). (C) The same scatterplot of *S2*/Spike-in ratio for each dilution sample as in (B), colored by template type (RNA in purple, viral particle in green).

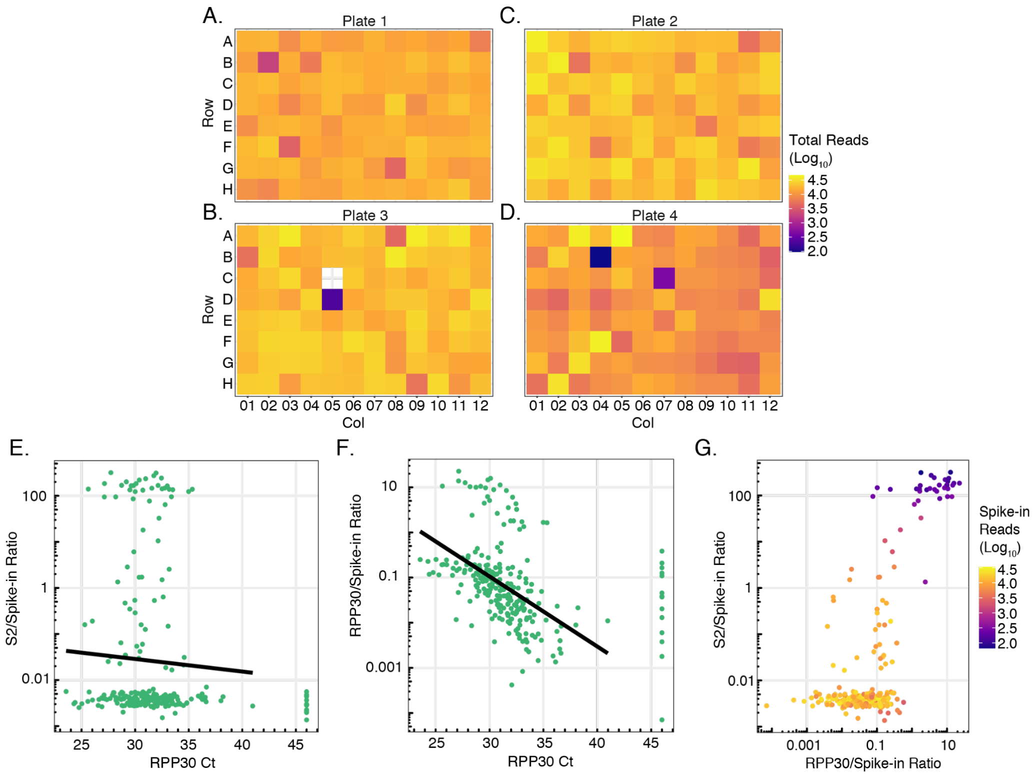

Figure S3. (A-D) Heatmaps of $\log_{10}$(total read depth) for every extracted RNA sample (four 96-well plates). (E) Scatterplot of *RPP30* Ct value from qRT-PCR (x-axis) compared to the *S2*/Spike-in ratio from Swab-

Seq (y-axis). (F) Scatterplot of *RPP30* Ct value from qRT-PCR (x-axis) compared to the *RPP30*/Spike-in ratio from Swab-Seq (y-axis). (G) Scatterplot of *RPP30*/Spike-in ratio from Swab-Seq (x-axis) compared to the *S2*/Spike-in ratio from Swab-Seq (y-axis). Points are colored by the total number of Spike-in reads for that sample.

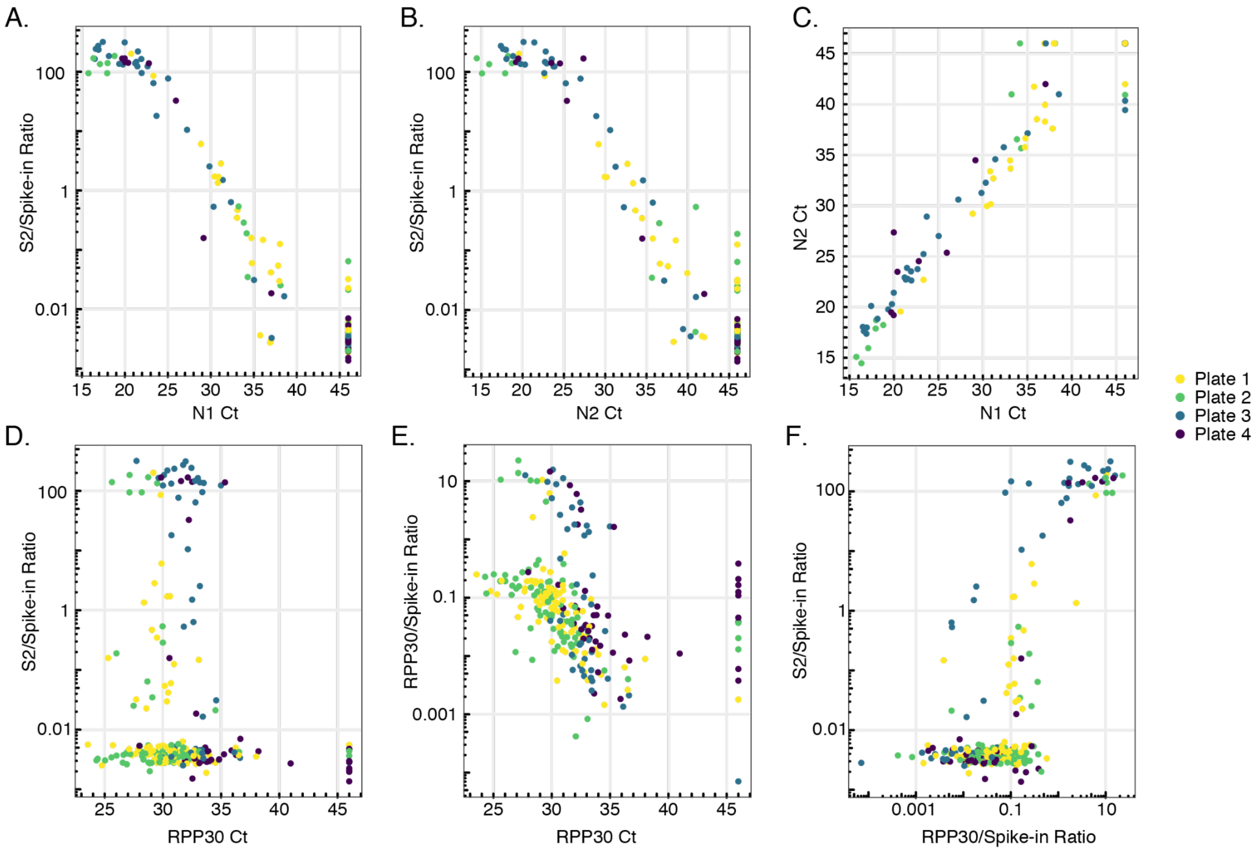

Figure S4. (A) Scatterplot of *N1* Ct value from qRT-PCR compared to *S2*/Spike-in ratio from Swab-Seq (y-axis) colored by sample plate. (B) Scatterplot of *N2* Ct value from qRT-PCR compared to *S2*/Spike-in ratio from Swab-Seq (y-axis) colored by sample plate. (C) Scatterplot of *N1* Ct value from qRT-PCR compared to *N2* Ct value from qRT-PCR (y-axis) colored by sample plate. (D) Scatterplot of *RPP30* Ct value from qRT-PCR compared to *S2*/Spike-in ratio (y-axis) colored by sample plate. (E) Scatterplot of *RPP30* Ct from qRT-PCR value compared to *RPP30*/Spike-in ratio from Swab-Seq (y-axis) colored by sample plate. (F) Scatterplot of *RPP30*/Spike-in ratio from Swab-Seq compared to *S2*/Spike-in ratio from Swab-Seq (y-axis) colored by sample plate.

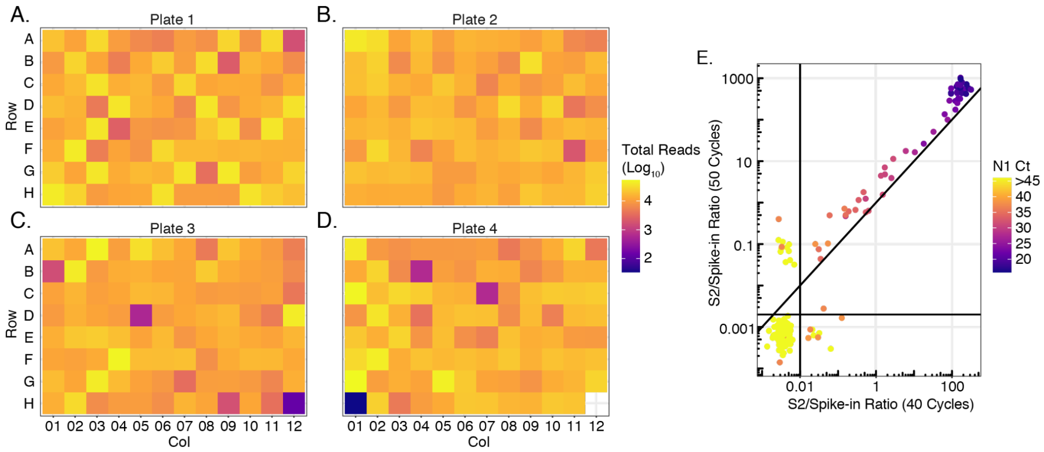

Figure S5. (A-D) Heatmaps of $\log_{10}$(total read depth) for every extracted RNA sample (four 96-well plates) after amplifying for 50 cycles. (E) Scatterplot of *S2*/Spike-in ratio for 40 cycle Swab-Seq compared to *S2*/Spike-in ratio for 50 cycle Swab-Seq for subject samples. Color of points indicate Ct value from qRT-PCR for that sample. Yellow points were undetected. Blue through orange colors indicate a qRT-PCR Ct value less than 40.

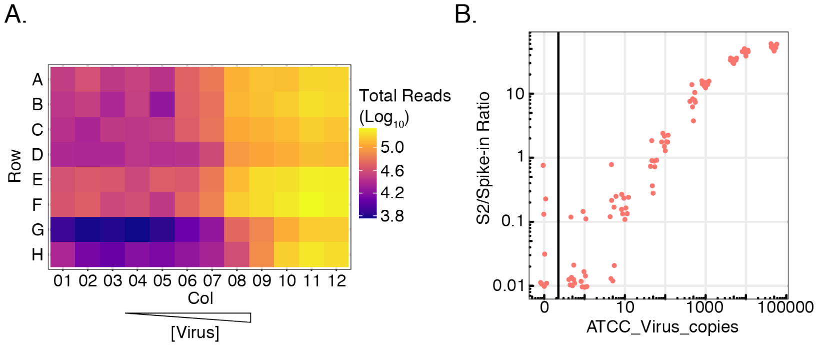

Figure S6. (A) Heatmap of $\log_{10}$(total read depth) for all samples for the serial dilution in saliva. Columns represent increasing concentrations of virus, from 0 copies in column 1 to 50,000 copies in column 12. (B) Data points for number of loaded particles of inactivated virus (x-axis) and *S2*/Spike-in ratio (y-axis) without superimposed linear trends for the same comparison.

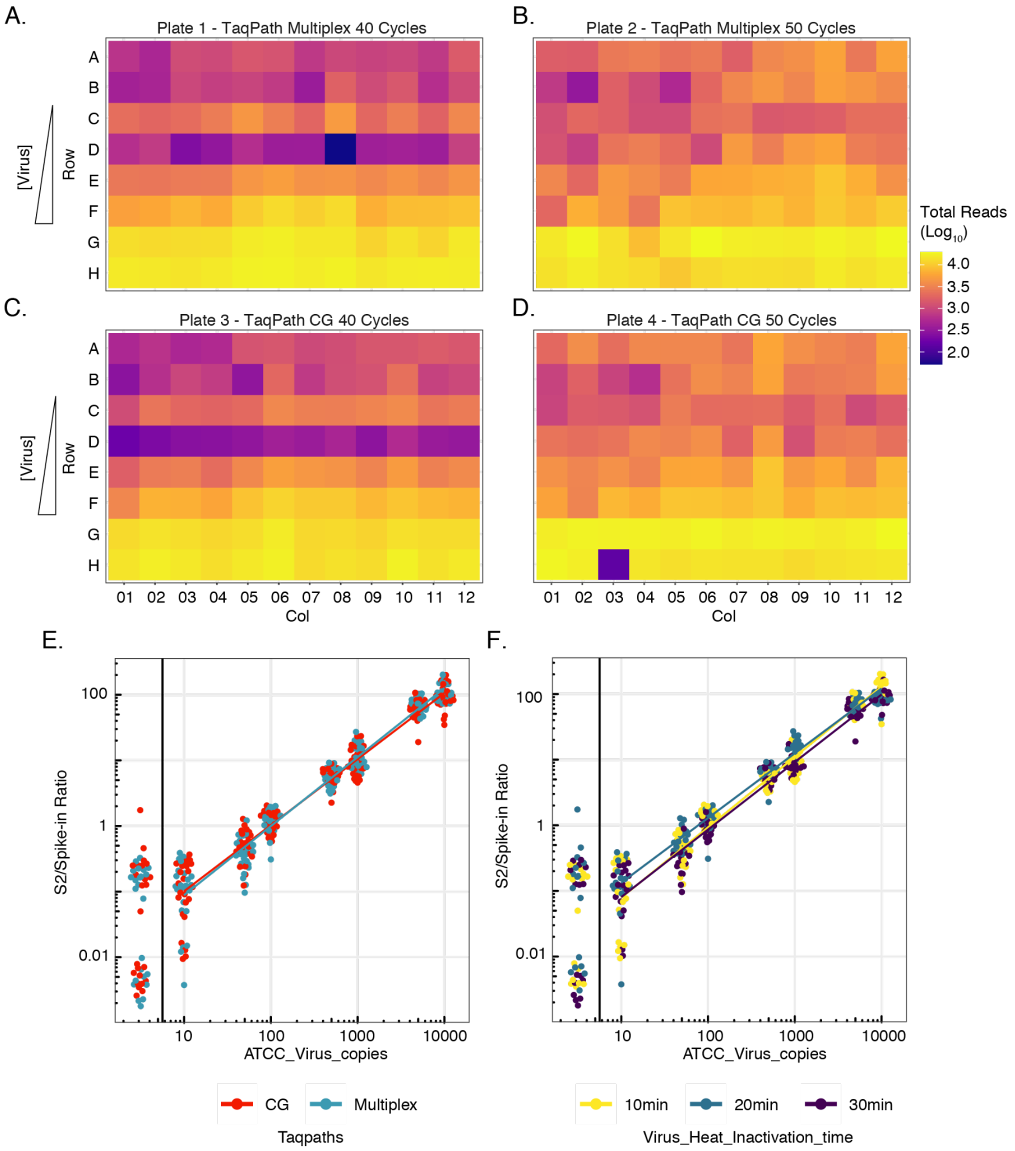

Figure S7. (A-D) Heatmaps of $\log_{10}$(total read depth) for serial dilutions used to test various conditions in saliva. For each plate, columns 1-4 were inactivated for 10 min, columns 5-8 were inactivated for 20 min, and columns 9-12 were inactivated for 30 min (all at 95°C). Rows represent increasing concentrations of virus, from 0 copies in row A to 10,000 copies in row H. (E) Scatterplot of *S2*/Spike-in ratio for each dilution sample colored by TaqPath kit used. (F) Scatterplot of *S2*/Spike-in ratio for each dilution sample colored by inactivation time.

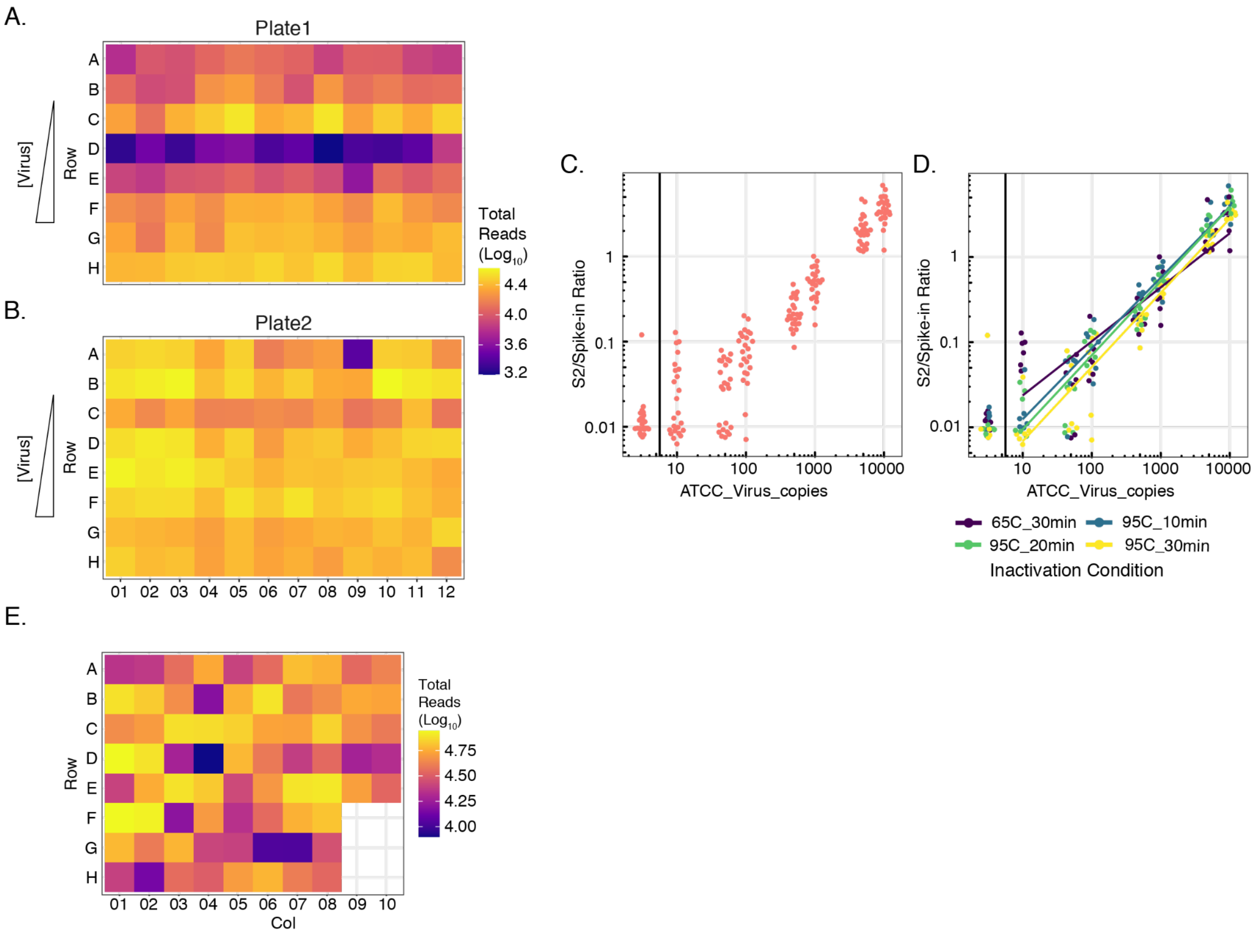

Figure S8. (A-B) Heatmaps of $\log_{10}$(total read depth) for serial dilutions used to test virus inactivation conditions in saline gargle. For Plate 1 (A), columns 1-6 were inactivated at 65°C for 30 min and columns 7-12 were inactivated at 95°C for 10 min. For Plate 2 (B), columns 1-6 were inactivated at 95°C for 20 min and columns 7-12 were inactivated at 95°C for 30 min. Rows represent increasing concentrations of virus, from 0 copies in row A to 10,000 copies in row H. (C) Scatterplot of *S2*/Spike-in ratio for each dilution sample. (D) The same scatterplot of *S2*/Spike-in ratio for each dilution sample as in (C), colored by inactivation condition. (E) Heatmap of $\log_{10}$(total read depth) for saline gargle samples from subjects.

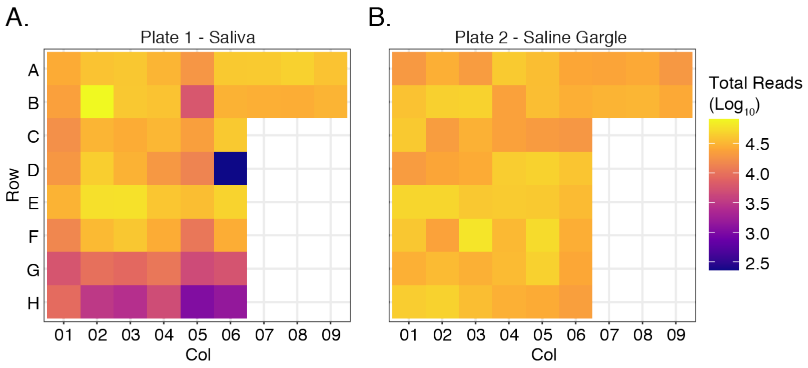

Figure S9. (A-B) Heatmaps of $\log_{10}$(total read depth) for samples profiled in the stability test. Plate 1 includes the saliva samples. Plate 2 includes the saline gargle samples.

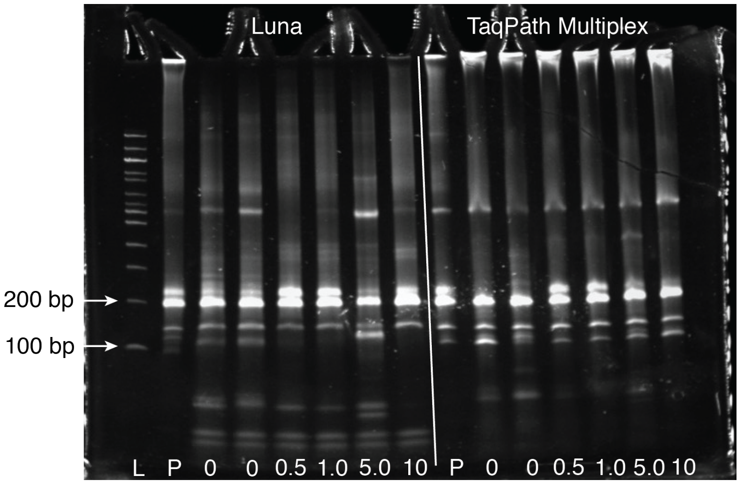

Figure S10. Representative gel of PCR products from the Luna vs TaqPath Multiplex comparison. The *S2* amplicon band is at ~200 bp. The *RPP30* amplicon band is at ~150 bp. Many other bands can be apparent, including primer dimers. Each lane is an individual sample. “L” - ladder; “P” - pool of samples; numbers 0-10 indicate copies of viral RNA (x 1,000) in reaction loaded on that lane.

Supplementary Table Legends

NOTE: Supplementary tables are too large to be included easily in one document and so are provided as separate files. Here we provide the legends for reference.

Table S1. Results for Luna vs Taqpath. The table includes the following columns:

Column 1: “Index” - The combined i7 and i5 barcodes used to address a particular well.

Column 2: “Index1” - The i7 index for each sample.

Column 3: “Index2” - The i5 index for each sample.

Column 4: “Plate_ID” - The name of the plate(s) used in an experiment.

Column 5: “Sample_Well” - The well of the sample on a plate. Columns are 1-12, rows are A-H.

Column 6: “Sample_ID” - Plate_ID and Sample_Well concatenated together.

Column 7: “Twist_RNA_copies” - The number of RNA copies loaded into each well (ranging from 0 to 10,000).

Column 8: “Enzyme” - The 1-step enzyme used (“Luna” or “TaqPath”).

Column 9: “S2_Count” - The number of reads mapping to *S2* for that barcode.

Column 10: “Spike_Count” - The number of reads mapping to the spike-in control for that barcode.

Column 11: “RPP30_Count” - The number of reads mapping to *RPP30* for that barcode.

Column 12: “S2_Spike_Ratio” - The *S2*/Spike-in ratio for that sample, calculated as (S2_Count + 1)/(Spike_Count + 1).

Table S2. Results for TaqPath Multiplex vs Taqpath CG. The table includes the following columns:

Column 1: “Index” - The combined i7 and i5 barcodes used to address a particular well.

Column 2: “Index1” - The i7 index for each sample.

Column 3: “Index2” - The i5 index for each sample.

Column 4: “Plate_ID” - The name of the plate(s) used in an experiment.

Column 5: “Sample_Well” - The well of the sample on a plate. Columns are 1-12, rows are A-H.

Column 6: “Sample_ID” - Plate_ID and Sample_Well concatenated together.

Column 7: “Twist_RNA_copies” - The number of RNA copies loaded into each well (ranging from 0 to

10,000).

Column 8: “Plate_config” - The 1-step enzyme used (“Multiplex” or “CG”).

Column 9: “S2_Count” - The number of reads mapping to *S2* for that barcode.

Column 10: “Spike_Count” - The number of reads mapping to the spike-in control for that barcode.

Column 11: “RPP30_Count” - The number of reads mapping to *RPP30* for that barcode.

Column 12: “S2_Spike_Ratio” - The *S2*/Spike-in ratio for that sample, calculated as (S2_Count + 1)/(Spike_Count + 1).

Table S3. Results for RNA vs viral particle template. The table includes the following columns:

Column 1: “Index” - The combined i7 and i5 barcodes used to address a particular well.

Column 2: “Index1” - The i7 index for each sample.

Column 3: “Index2” - The i5 index for each sample.

Column 4: “Plate_ID” - The name of the plate(s) used in an experiment.

Column 5: “Sample_Well” - The well of the sample on a plate. Columns are 1-12, rows are A-H.

Column 6: “Sample_ID” - Plate_ID and Sample_Well concatenated together.

Column 7: “RNA_virus_copies” - The number of RNA copies loaded into each well (ranging from 0 to 10,000).

Column 8: “Plate_config” - The template used (“RNA” or “Virus”).

Column 9: “S2_Count” - The number of reads mapping to *S2* for that barcode.

Column 10: “Spike_Count” - The number of reads mapping to the spike-in control for that barcode.

Column 11: “RPP30_Count” - The number of reads mapping to *RPP30* for that barcode.

Column 12: “S2_Spike_Ratio” - The *S2*/Spike-in ratio for that sample, calculated as (S2_Count + 1)/(Spike_Count + 1).

Table S4. Results for RNA samples. The table includes the following columns:

Column 1: “Index” - The combined i7 and i5 barcodes used to address a particular well.

Column 2: “Index1” - The i7 index for each sample.

Column 3: “Index2” - The i5 index for each sample.

Column 4: “Plate_ID” - The name of the plate(s) used in an experiment.

Column 5: “Sample_Well” - The well of the sample on a plate. Columns are 1-12, rows are A-H.

Column 6: “Sample_ID” - Plate_ID and Sample_Well concatenated together.

Column 7: “Plate_config” - A unique identifier for each sample. Either starts with “ASYMPT” (a population of asymptomatic individuals), “SYMPT” (a population of symptomatic individuals), “NEG” (negative control wells), “EXT” (human cell extraction control), “NPOS” (control sample that contains sequence from the *N* gene of SARS-CoV-2), and “SPOS” (control sample that contains sequence from the *S* gene of SARS-CoV-2).

Column 8: “Ct_N1” - Ct value (average of triplicates) for qPCR using the CDC *N1* primer/probe set. Samples with no detectable Ct are labelled “Undetermined”.

Column 9: “Ct_N2” - Ct value (average of triplicates) for qPCR using the CDC *N2* primer/probe set. Samples with no detectable Ct are labelled “Undetermined”.

Column 10: “Ct_RPP30” - Ct value (average of triplicates) for qPCR using the CDC *RNaseP* primer/probe set. Samples with no detectable Ct are labelled “Undetermined”.

Column 11: “S2_Count” - The number of reads mapping to *S2* for that barcode.

Column 12: “Spike_Count” - The number of reads mapping to the spike-in control for that barcode.

Column 13: “RPP30_Count” - The number of reads mapping to *RPP30* for that barcode.

Column 14: “S2_Spike_Ratio” - The *S2*/Spike-in ratio for that sample, calculated as (S2_Count + 1)/(Spike_Count + 1).

Table S5. Results for RNA samples 50 cycles. The table includes the following columns:

Column 1: “Index” - The combined i7 and i5 barcodes used to address a particular well.

Column 2: “Index1” - The i7 index for each sample.

Column 3: “Index2” - The i5 index for each sample.

Column 4: “Plate_ID” - The name of the plate(s) used in an experiment.

Column 5: “Sample_Well” - The well of the sample on a plate. Columns are 1-12, rows are A-H.

Column 6: “Sample_ID” - Plate_ID and Sample_Well concatenated together.

Column 7: “Plate_config” - A unique identifier for each sample. Either starts with “ASYMPT” (a population

of asymptomatic individuals), “SYMPT” (a population of symptomatic individuals), “NEG” (negative control wells), “EXT” (human cell extraction control), “NPOS” (control sample that contains sequence from the *N* gene of SARS-CoV-2), and “SPOS” (control sample that contains sequence from the *S* gene of SARS-CoV-2).

Column 8: “Ct_N1” - Ct value (average of triplicates) for qPCR using the CDC *N1* primer/probe set. Samples with no detectable Ct are labelled “Undetermined”.

Column 9: “Ct_N2” - Ct value (average of triplicates) for qPCR using the CDC *N2* primer/probe set. Samples with no detectable Ct are labelled “Undetermined”.

Column 10: “Ct_RPP30” - Ct value (average of triplicates) for qPCR using the CDC *RNaseP* primer/probe set. Samples with no detectable Ct are labelled “Undetermined”.

Column 11: “S2_Count” - The number of reads mapping to *S2* for that barcode.

Column 12: “Spike_Count” - The number of reads mapping to the spike-in control for that barcode.

Column 13: “RPP30_Count” - The number of reads mapping to *RPP30* for that barcode.

Column 14: “S2_Spike_Ratio” - The *S2*/Spike-in ratio for that sample, calculated as (S2_Count + 1)/(Spike_Count + 1).

Table S6. Results for saliva dilutions in 96-well vs 384-well plates. The table includes the following columns:

Column 1: “Index” - The combined i7 and i5 barcodes used to address a particular well.

Column 2: “Index1” - The i7 index for each sample.

Column 3: “Index2” - The i5 index for each sample.

Column 4: “Plate_ID” - The name of the plate(s) used in an experiment.

Column 5: “Sample_Well” - The well of the sample on a plate. Columns are 1-12, rows are A-H.

Column 6: “Sample_ID” - Plate_ID and Sample_Well concatenated together.

Column 7: “ATCC_Virus_copies” - The number of heat-inactivated viral particles loaded into each well (ranging from 0 to 10,000).

Column 8: “Plate_config” - Whether the sample was cycled on a 96-well or 384-well plate (“96well” or “384well”).

Column 9: “S2_Count” - The number of reads mapping to *S2* for that barcode.

Column 10: “Spike_Count” - The number of reads mapping to the spike-in control for that barcode.

Column 11: “RPP30_Count” - The number of reads mapping to *RPP30* for that barcode.

Column 12: “S2_Spike_Ratio” - The *S2*/Spike-in ratio for that sample, calculated as (S2_Count + 1)/(Spike_Count + 1).

Table S7. Results for saliva dilutions in various conditions. The table includes the following columns:

Column 1: “Index” - The combined i7 and i5 barcodes used to address a particular well.

Column 2: “Index1” - The i7 index for each sample.

Column 3: “Index2” - The i5 index for each sample.

Column 4: “Plate_ID” - The name of the plate(s) used in an experiment.

Column 5: “Sample_Well” - The well of the sample on a plate. Columns are 1-12, rows are A-H.

Column 6: “Sample_ID” - Plate_ID and Sample_Well concatenated together.

Column 7: “ATCC_Virus_copies” - The number of heat-inactivated viral particles loaded into each well (ranging from 0 to 10,000).

Column 8: “Taqpaths” - Taqpath formulation tested (“Multiplex” or “CG”).

Column 9: “Virus_Heat_Inactivation_time” - The duration of time at 95C before dilution in TBE and Tween-20 (“10min”, “20min”, or “30min”).

Column 10: “Cycle_numbers” - The number of cycles of amplification (“40cycles” or “50cycles”).

Column 11: “S2_Count” - The number of reads mapping to *S2* for that barcode.

Column 12: “Spike_Count” - The number of reads mapping to the spike-in control for that barcode.

Column 13: “RPP30_Count” - The number of reads mapping to *RPP30* for that barcode.

Column 14: “S2_Spike_Ratio” - The *S2*/Spike-in ratio for that sample, calculated as (S2_Count + 1)/(Spike_Count + 1).

Table S8. Results for gargle inactivation conditions. The table includes the following columns:

Column 1: “Index” - The combined i7 and i5 barcodes used to address a particular well.

Column 2: “Index1” - The i7 index for each sample.

Column 3: “Index2” - The i5 index for each sample.

Column 4: “Plate_ID” - The name of the plate(s) used in an experiment.

Column 5: “Sample_Well” - The well of the sample on a plate. Columns are 1-12, rows are A-H.

Column 6: “Sample_ID” - Plate_ID and Sample_Well concatenated together.

Column 7: “ATCC_Virus_copies” - The number of heat-inactivated viral particles loaded into each well (ranging from 0 to 10,000).

Column 8: “Plate_config” - The inactivation condition tested (“65C_30min”, “95C_10min”, “95C_20min” or “95C_30min”).

Column 9: “S2_Count” - The number of reads mapping to *S2* for that barcode.

Column 10: “Spike_Count” - The number of reads mapping to the spike-in control for that barcode.

Column 11: “RPP30_Count” - The number of reads mapping to *RPP30* for that barcode.

Column 12: “S2_Spike_Ratio” - The *S2*/Spike-in ratio for that sample, calculated as (S2_Count + 1)/(Spike_Count + 1).

Table S9. Results for gargle samples from subjects. The table includes the following columns:

Column 1: “Index” - The combined i7 and i5 barcodes used to address a particular well.

Column 2: “Index1” - The i7 index for each sample.

Column 3: “Index2” - The i5 index for each sample.

Column 4: “Plate_ID” - The name of the plate(s) used in an experiment.

Column 5: “Sample_Well” - The well of the sample on a plate. Columns are 1-12, rows are A-H.

Column 6: “Sample_ID” - Plate_ID and Sample_Well concatenated together.

Column 7: “Plate_samples” - The sample ID for the individual.

Column 8: “Replicates” - Indication of whether the sample was part of replicate set 1 or 2 (“rep1” or “rep2”).

Column 9: “Ct_N1” - Ct value (average of triplicates) for qPCR using the CDC *N1* primer/probe set. Samples with no detectable Ct are labelled “Undetermined”.

Column 10: “Ct_RPP30” - Ct value (average of triplicates) for qPCR using the CDC *RNaseP* primer/probe set. Samples with no detectable Ct are labelled “Undetermined”.

Column 11: “S2_Count” - The number of reads mapping to *S2* for that barcode.

Column 12: “Spike_Count” - The number of reads mapping to the spike-in control for that barcode.

Column 13: “RPP30_Count” - The number of reads mapping to *RPP30* for that barcode.

Column 14: “S2_Spike_Ratio” - The *S2*/Spike-in ratio for that sample, calculated as (S2_Count + 1)/(Spike_Count + 1).

Table S10. Results for stability test. The table includes the following columns:

Column 1: “Index” - The combined i7 and i5 barcodes used to address a particular well.

Column 2: “Index1” - The i7 index for each sample.

Column 3: “Index2” - The i5 index for each sample.

Column 4: “Plate_ID” - The name of the plate(s) used in an experiment.

Column 5: “Sample_Well” - The well of the sample on a plate. Columns are 1-12, rows are A-H.

Column 6: “Sample_ID” - Plate_ID and Sample_Well concatenated together.

Column 7: “ATCC_Virus_copies” - The number of heat-inactivated viral particles loaded into each well (ranging from 0 to 10,000).

Column 8: “Subjects” - The subject ID for each individual donating saliva or saline gargle sample (“Subject1”, “Subject2”, or “Subject3”).

Column 9: “Storage_condition” - The type of storage for each sample (“freezer”, “RT”, or “None”). “None” indicates that the sample was heat-inactivated on the day it was collected.

Column 10: “Storage_duration” - The number of days the sample was stored (“0”, “1”, “7”, or “-1.75”). “-1.75” is a placeholder for the negative control samples that did not receive viral particles and were heat-inactivated on the day they were collected.

Column 11: “Saliva_vs_Gargle” - Indicator whether the sample was saliva or saline gargle (“Saliva” or “Gargle”).

Column 12: “S2_Count” - The number of reads mapping to *S2* for that barcode.

Column 13: “Spike_Count” - The number of reads mapping to the spike-in control for that barcode.

Column 14: “RPP30_Count” - The number of reads mapping to *RPP30* for that barcode.

Column 15: “S2_Spike_Ratio” - The *S2*/Spike-in ratio for that sample, calculated as (S2_Count + 1)/(Spike_Count + 1).

Table S11. Sequencing results for each experiment. The table includes the following columns:

Column 1: “Experiment” - The name of the experiment sequenced.

Column 2: “Most Relevant Figure” - The main figure that shows results from this experiment.

Column 3: “Perfect Match BC” - The number of barcodes that perfectly matched an expected barcode from this experiment when demultiplexing the sequencing run.

Column 4: “Edited BC” - The number of barcodes that could be matched by allowing a hamming distance of 1 to the expected barcodes.

Column 5: “Total Filtered Reads” - The total number of perfectly matching and single error barcode reads that could be demultiplexed for this experiment.

Column 6: “Invalid BCs Observed” - The number of invalid barcode combinations observed while demultiplexing reads. By “invalid”, we mean the individual i5 and i7 barcodes were represented in the experiment, but the combination was not. These are indicative of a barcode swapping event.

Column 7: “N” - The number of PCR wells included in this experiment.

Column 8: “Reads/Sample” - The number of reads dedicated to each PCR well on average for this experiment. Calculated as “Total Filtered Reads” / “N”.

Column 9: “Sequencer Used” - Indicates whether the library was sequenced on a MiSeq or a NextSeq sequencer. If sequenced on a MiSeq, further specifies whether a 150 cycle v3 kit or a 50 cycle v2 kit was used.

Column 10: “Pooled with” - Indicates what other libraries the experiment was sequenced with. In some cases, the experiment was sequenced alone with the PhiX control (“PhiX”). In some cases, the experiment was pooled with other experiments and PhiX (“PhiX and other libraries”). In some cases, the experiment was pooled with libraries not related to Swab-Seq (“Other libraries”).

Table S12. Primer sequences used in this study. This table is a spreadsheet that contains multiple tabs detailing the sequences used. The file contains the following tabs:

“Primers for *S2* spike-in synth.” - Forward and reverse primers for amplifying cDNA of the *S2* region for *in vitro* synthesis.

"*S2* barcodes and primers" - For serial dilution experiments, we used combinatorial indexing. This tab gives the primer sequences and barcodes used for forward and reverse primers targeting the *S2* region. These primers amplify both the viral RNA and the spike-in control.

"RPP30 barcodes and primers" - For serial dilution experiments, we used combinatorial indexing. This tab gives the primer sequences and barcodes used for forward and reverse primers targeting the *RPP30* human control gene.

"Barcodes for Subject samples" - For experiments involving samples from subjects, we used unique dual indices. This tab indicates the barcodes that mark individual wells of 4 96-well (or 1 384-well plate). Both *S2* and *RPP30* used the same barcodes.

"Custom primers for sequencing" - In order to sequence these libraries, custom primers had to be loaded on the sequencer. This tab provides the sequences of those custom sequencing primers.
